# Supplementary material for: Key factors influencing motivation among health extension workers and health care professionals in four regions of Ethiopia: A cross-sectional study
Source: PLoS One. 2022 Sep 30;17(9):e0272551. doi: 10.1371/journal.pone.0272551 (PMC9524639; doi:10.1371/journal.pone.0272551)
Supplement: S1 Appendix — Results from ordered logit model showing factors associated with overall motivation among participants form four regions April 15- May10, 2018. (PDF) [file pone.0272551.s002.pdf]

Appendix 1

Table A1. Results from ordered logit model showing factors associated with overall motivation among participants form four regions April15-May10, 2018

Factor	Coef.	95% CI	P-value
Region			
Amhara	Reference		
Oromia	0.44	(-0.10, 0.99)	0.11
SNNPR	-0.84	(-1.41, -0.28)	0.003
Tigray	-0.11	(-0.72, 0.51)	0.74
Job title			
HEW	Reference		
Health care providers	0.36	(0.11, -0.05)	0.12
Leaders	-0.41	(-0.96, 0.12)	0.13
Other	-0.50	(-1.47, 0.47)	0.31
Workload			
Light: more than enough time to complete duties	Reference		
Medium: enough time to complete duties	-1.26	(-2.35, -0.18)	0.02
Heavy: barely enough time to complete duties	-0.78	(-1.84, 0.28)	0.15
Perceived gross salary fair			
Very fair	Reference		
Quite fair	0.29	(-1.66, 2.24)	0.29
Neither fair nor unfair	1.02	(-1.02, 3.07)	0.98

Quite unfair	0.50	(-1.46, 2.47)	0.50
Very unfair	0.17	(-1.86, 2.20)	0.16
Average job satisfaction	0.61	(0.32, 0.89)	0.001
