# Supplementary material for: Key factors influencing motivation among health extension workers and health care professionals in four regions of Ethiopia: A cross-sectional study
Source: PLoS One. 2022 Sep 30;17(9):e0272551. doi: 10.1371/journal.pone.0272551 (PMC9524639; doi:10.1371/journal.pone.0272551)
Supplement: S2 Appendix — Results from ordered logit model showing association between motivation factors and demographic and structural factors among participants from four regions April 15- May10,2018. (PDF) [file pone.0272551.s003.pdf]

Appendix2

## Table A2. Results from ordered logit model showing association between motivation factors and demographic and structural factors among participants from four regions April15- May10,2018.

| | Factor 1: personal and altruistic goals | | | Factor 2: pride and personal satisfaction | | | Factor 3: recognition and support | | |
|---|---|---|---|---|---|---|---|---|---|
| Factor | Coef. | 95% CI | P-value | Coef. | 95% CI | P-value | Coef. | 95% CI | P-value |
| Gender | | | | | | | | | |
| Male | Reference | | | Reference | | | Reference | | |
| Female | -0.19 | (-0.75, 0.37) | 0.50 | -0.13 | (-0.59, 0.32) | 0.56 | -0.26 | (-0.84, 0.32) | 0.378 |
| Region | | | | | | | | | |
| Amhara | Reference | | | Reference | | | Reference | | |
| Oromia | -0.80 | (-1.32, -0.27) | 0.003 | -0.13 | (-0.67, 0.41) | 0.63 | -0.56 | (-1.11, -0.02) | 0.043 |
| SNNPR | -0.68 | (-1.25, -0.11) | 0.019 | -0.53 | (-1.06, -0.015) | 0.04 | -0.62 | (-1.20, -0.05) | 0.031 |
| Tigray | -0.13 | (-0.73, 0.64) | 0.66 | 0.38 | (-0.20, 0.97) | 0.20 | 0.46 | (-1.55, 1.07) | 0.143 |
| Age | | | | | | | | | |
| 19-24 | Reference | | | Reference | | | Reference | | |
| 25-30 | -0.25 | (-0.70, 0.20) | 0.28 | 0.47 | (-0.03, 0.97) | 0.065 | -0.197 | (-0.71, 0.31) | 0.45 |
| >30 | 0.05 | (-0.65, 0.54) | 0.86 | 0.71 | (0.06, 1.36) | 0.032 | 0.098 | (-0.56, 0.76) | 0.77 |
| Perceived gross salary fair | | | | | | | | | |
| Very fair | Reference | | | | | | Reference | | |

| | | | | | | | | | |
|---|---|---|---|---|---|---|---|---|---|
| Quite fair | -1.44 | (-3.06, 0.11) | 0.07 | | | | -0.675 | (-2.35, 1.00) | 0.431 |
| Neither fair nor unfair | -1.45 | (-3.12, 0.18) | 0.08 | | | | 0.022 | (-1.72, 1.77) | 0.979 |
| Quite unfair | -1.14 | (-2.77, 0.40) | 0.15 | | | | 0.101 | (-1.58, 1.78) | 0.906 |
| Very unfair | -1.32 | (-3.0, 0.33) | 0.11 | | | | 0.337 | (-1.42, 2.09) | 0.706 |
| Work experience | | | | | | | | | |
| <6month | | | | Reference | | | | | |
| 6month-1year | | | | -0.129 | (-0.86, 0.60) | 0.728 | -0.47 | (-1.25, 0.31) | 0.236 |
| 1-2years | | | | -0.025 | (-0.74, 0.69) | 0.945 | -0.12 | (-0.86, 0.62) | 0.756 |
| 2-4years | | | | 0.364 | (-0.29, 1.02) | 0.279 | -0.39 | (-1.10, 0.31) | 0.275 |
| >4years | | | | -0.275 | (-0.85, 0.30) | 0.349 | -0.36 | (-0.98, 0.26) | 0.256 |
| Job title | | | | | | | | | |
| HEW | | | | | | | Reference | | |
| Healthcare provides | | | | | | | 0.47 | (-0.001, 0.95) | 0.051 |
| Leaders | | | | | | | 0.76 | (-0.64, 0.79) | 0.83 |
| Other | | | | | | | -0.19 | (-1.18, 0.79) | 0.70 |
| Average job satisfaction | 1.98 | (1.64, 2.34) | 0.001 | 2.94 | (2.53, 3.34) | 0.001 | 1.65 | (1.35, 1.95) | 0.001 |
| Average leave | -0.34 | (-0.55, -0.13) | 0.002 | -0.25 | (-0.45, -0.05) | 0.013 | -0.31 | (-0.52, -0.12) | 0.003 |
