# Supplementary material for: Key factors influencing motivation among health extension workers and health care professionals in four regions of Ethiopia: A cross-sectional study
Source: PLoS One. 2022 Sep 30;17(9):e0272551. doi: 10.1371/journal.pone.0272551 (PMC9524639; doi:10.1371/journal.pone.0272551)
Supplement: Attachment — Submitted filename: cheru comment.pdf [file pone.0272551.s007.pdf]

PLOS ONE

Key factors influencing motivation among health extension workers and health care professionals in four regions of Ethiopia: a cross-sectional study.

--Manuscript Draft--

**Manuscript Number:**	PONE-D-21-09235R2
**Article Type:**	Research Article
**Full Title:**	Key factors influencing motivation among health extension workers and health care professionals in four regions of Ethiopia: a cross-sectional study.
**Short Title:**	Key factors influencing motivation among health extension workers and health care professionals in Ethiopia
**Corresponding Author:**	Mehiret Abate Adillo, MPH<br>Institute for Healthcare Improvement<br>Addis Ababa, ETHIOPIA
**Keywords:**	Motivation, healthcare workers, health extension workers
**Abstract:**	Abstract<br><br>Background Although Ethiopia has improved access to health care in recent years, quality of care remains low. Health worker motivation is an important determinant of performance and affects quality of care. Low health care workers motivation can be associated with poor health care quality and client experience, long patient waiting time, non-attendance, and poor clinical outcome. Objective this study sought to determine the extent and variation of health professionals' motivation alongside factors associated with motivation. Methods We conducted a facility based cross-sectional study among health extension workers (HEWs) and health care professionals in four regions: Amhara, Oromia, South nations, and nationalities people's region (SNNPR) and Tigray from April 15 to May 10, 2018. We sampled 401 health system workers: skilled providers including nurses and midwives (n=110), HEWs (n=210); and non-patient facing health system staff representing case team leaders, facility and district heads, directors, and officers (n=81). Participants completed a 30-item Likert scale ranking tool which asked questions across 17 domains. We used exploratory factor analysis to explore latent motivation constructs. Results Of the 397 responses with complete data, 61% (95% CI 56%-66%) self-reported motivation as "very good" or "excellent". Significant variation in motivation was seen across regions with SNNPR scoring significantly lower on a five-point Likert scale by 0.35 points (P=0.003). The exploratory factor analysis identified a three-factors: personal and altruistic goals; pride and personal satisfaction; and recognition and support. The personal and altruistic goals factor varied across regions with Oromia and SNNPR being significantly lower by 0.13 (P=0.018) and 0.12 (P=0.039) Likert points respectively. The pride and personal satisfaction factor were higher among those aged >=30 years by 0.14 Likert scale points (P=0.045) relative to those aged between 19-24years. Conclusions Overall, motivation was high among participants but varied across region, cadre, and age. Workload, leave, and job satisfaction were associated with motivation.
**Order of Authors:**	Mehiret Abate Adillo, MPH
	Zewdie Mulisa
	Hema Magge
	Befikadu Bitewulign Desta
	Abiyou Kiflie Alemayehu
	Abera Biadgo Kefale
	Dorka Woldesenbet Keraga
	Haregeweyni Alemu Gebreselassie
	Yakob Seman Ahmed
	Abiy Seifu Estifanos
	Gareth Parry

Powered by Editorial Manager® and ProduXion Manager® from Aries Systems Corporation

	Matthew Quaife
**Response to Reviewers:**	Feb 16, 2022<br>Manuscript PONE-D-21-09235<br>Response to reviewers<br>Dear Editor in Chief<br>Thank you so much for the opportunity you have given us to re-submit a revised manuscript entitled “Key factors influencing motivation among health extension workers and health care professionals in four regions of Ethiopia: a cross sectional study.” for consideration for publication in PLOS ONE.<br>We appreciate the time and effort you and the reviewers dedicated to providing feedback to our manuscript and are grateful for the insightful comments on and valuable improvements made to our paper.<br>We have incorporated most of the suggestions made by the reviewer. Those changes are highlighted with yellow color within the revised manuscript with track changes. Please see below a blue colored point-by-point response to the reviewers’ comments and concerns. Please note that all page numbers and line numbers refer to the revised manuscript.<br>Thank you so much once again!<br>Reviewer #1 no comment<br>Reviewer# 2: Key factors influencing motivation among health extension workers and health care professionals in four regions of Ethiopia: a cross-sectional study.<br>I am excited to get the opportunity to review the manuscript mentioned above. In general, the manuscript is a well-stated and essential area for the developing country. However, before accepting publication, the authors should consider the following issues.<br><br>Abstract:<br>Include the objective of the study in the abstract section. Make sure to use the same abstract in the system and main manuscript. The abstract in the main manuscript has no objectives, while the abstract in the system contains it.<br>Authors Response: thank you for pointing out this. We have included the objective on page 2 line 26-27 and it reads as ’Objective this study sought to determine the extent and variation of health professionals’ motivation alongside factors associated with motivation.’<br>What does non-patient-facing health system staff mean? Who are they?<br>Authors response: thank you for asking, non-patient facing health system staffs are professionals working in different structures of a health system. It is stated on the abstract on page 2 line 32-33 as ’non-patient facing health system staff representing case team leaders, facility and district heads, directors, and officers (n=81).’<br>Please give space between a statement and citation throughout the manuscript.<br>Authors response: Thank you for the comment, corrected accordingly as shown on line 50, 53, 55, 65, 72, 106, 109, 188, 322, 325, 332 and 343.<br>Line 65 -66<br>What was the finding of the study? Why did the author explain these studies?<br>Authors response: Thank you, the study assessed motivation and retention of health care workers and indicated importance of financial incentives on health workers motivation. In our study, there are different factors associated with motivation including financial incentives and revealed that financial and managerial support were significantly associated with health professionals job satisfaction, region, leave days and job title as specified on the result section on page 22 line 300 -310. These also further explained on the discussion section on page 24 on line 341 - 343 as ‘Recognition and support were significantly higher among health care providers than among HEWs. Having more recognition (financial and managerial) support was a positive predictor for job satisfaction and motivation.’<br>Even though the sample size is smaller, it includes four regions (Amhara, Oromo, SNNPR, and Tigray), which is good and improves the representativeness of the finding. But, what is your justification for including these? Why not Harari, Gambella.<br>Authors response: we believed that the four regions are home to more than 81% of the population in Ethiopia and accommodates most health facilities and health workers. These regions are highly diverse population and have different geographic characteristics improving representativeness as stated on page 6, line number 100 - 102.<br>Table 5:

	Association between motivation factors and 271 demographic and structural factors<br>Use consistent decimal numbers throughout the document. Example region: (-0.21, 0.006) 0.064<br>Authors response: Thank you for pointing this out, the decimal numbers corrected accordingly throughout the document. On page number 13 table 2 (region _Amhara %), on page number 17-line number 249, on page number 18 Table 4 perceived gross salary fair (p value), on page number 19 in line number 270, on page number 20-21 table 5 gender factor 2 (coef.), region factor 2 (coef.), factor 2 region (95%CI), age>30 factor1 (95%CI), work experience factor2 (coef.), factor3 job title (95%CI), on page number 22 in line number 299.<br>Discussion session<br>Page 22 and line 301-302: add the figure with confidence interval.<br>Authors response: thank you for pointing out, we have included the interval accordingly it reads as 'the mean score of recognition and support was 1.77 (95% CI 1.72,1.83; P=0.001)'on page 22 line 299.<br>Page 22 and line 303: delete one "factor".<br>Authors response: thank you deleted accordingly.<br>Page 22 and lines 303 - 314: How do you think your finding is different from the previous one? Consider this comment for all results?<br>Authors response: line number 303-314 our study finding "motivation varied from region to region'' is different in its scope that the study was conducted in four big regions and regional variation of motivation was studied at the same time. In addition, the health care workers motivation and health extension workers motivation were compared, and this study is different in computing multiple characteristics with the overall motivation and three motivation factors.<br>The three factors also indicated variation with associated factors (region, age, leave days, job satisfaction, perceived gross salary, workload…) as on page number 17-line number 249-257, on page number 19-line number 271-280, and on page number 22-line number 287 – 295.<br>This has been addressed in the manuscript discussion section on page 24 and line number 345-349 stated as 'A strength of this study was that data was collected from four (Amhara, Oromia, SNNPR, Tigray) major regions of Ethiopia where most of the country's population live and the regional variation of motivation was studied at the same time, and the health care workers and health extension workers motivation were compared, in addition this study addressed computing multiple associated factors with the overall motivation and three motivation factors identified during factor analysis.'<br>What is the limitation of your study?<br>Authors response: thank you for this question, we have already stated a number of limitations in the manuscript on page24 & 25, line number 348-361:<br>'Motivation was self-reported and may therefore be subject to acceptability biases in the face-to-face interviews conducted. Knowledge that interviews were conducted by interviewers from a public university and evaluating an IHI programme may have biased responses. The skilled care provider sample was too small to conduct a subgroup analysis among specific groups of interest such as doctors or midwives. Region-level analyses may have been under powered to variation in motivation, particularly in the presence of heterogeneity in motivation within regions, for example by cadre, or other unobserved variables. The sampling strategy was not optimized to account for or detect potentially important heterogeneity between regions, urban and rural areas, or QI programme areas, which may further reduce power to detect across-region variation. Qualitative work could have been conducted alongside quantitative data collection to understand why variation exists in motivation between regions and personal characteristics. Finally, we were unable to assess the link between health worker motivation and the quality of care provided which remains under-researched.'
Additional Information:	
Question	Response
**Financial Disclosure**<br><br>Enter a financial disclosure statement that describes the sources of funding for the work included in this submission. Review the submission guidelines for detailed	This work was supported by IDEAS—Informed Decisions for Actions to improve maternal and newborn health (http://ideas.lshtm.ac.uk), which is funded through a grant from the Bill & Melinda Gates Foundation to the London School of Hygiene & Tropical Medicine. (Gates Global Health Grant Number: OPP1149259). The funder of this study had no role in the study's design or conduct, data collection, analysis or interpretation of results, writing of the paper, or decision to submit for publication

Powered by Editorial Manager® and ProduXion Manager® from Aries Systems Corporation

requirements. View published research articles from *PLOS ONE* for specific examples.<br><br>This statement is required for submission and **will appear in the published article** if the submission is accepted. Please make sure it is accurate.<br><br>**Unfunded studies**<br>Enter: *The author(s) received no specific funding for this work.*<br><br>**Funded studies**<br>Enter a statement with the following details:<br>• Initials of the authors who received each award<br>• Grant numbers awarded to each author<br>• The full name of each funder<br>• URL of each funder website<br>• Did the sponsors or funders play any role in the study design, data collection and analysis, decision to publish, or preparation of the manuscript?<br>• **NO** - Include this sentence at the end of your statement: *The funders had no role in study design, data collection and analysis, decision to publish, or preparation of the manuscript.*<br>• **YES** - Specify the role(s) played.<br><br>* typeset	
**Competing Interests**<br><br>Use the instructions below to enter a competing interest statement for this submission. On behalf of all authors, disclose any competing interests that could be perceived to bias this work—acknowledging all financial support and any other relevant financial or non-financial competing interests.<br><br>This statement **will appear in the published article** if the submission is accepted. Please make sure it is accurate. View published research articles from *PLOS ONE* for specific examples.	The authors have declared that no competing interests exist.

<table>
<tr>
<td>

NO authors have competing interests

Enter: *The authors have declared that no competing interests exist.*

Authors with competing interests

Enter competing interest details beginning with this statement:

I have read the journal's policy and the authors of this manuscript have the following competing interests: [insert competing interests here]

* typeset

</td>
<td></td>
</tr>
<tr>
<td>

Ethics Statement

Enter an ethics statement for this submission. This statement is required if the study involved:

- Human participants
- Human specimens or tissue
- Vertebrate animals or cephalopods
- Vertebrate embryos or tissues
- Field research

Write "N/A" if the submission does not require an ethics statement.

General guidance is provided below. Consult the submission guidelines for detailed instructions. **Make sure that all information entered here is included in the Methods section of the manuscript.**

</td>
<td>

Written informed consent was obtained from all participants. The study was undertaken with ethical approval from the Observational Research Ethics Committee of the London School of Hygiene and Tropical Medicine (Ref: 14429) and a program evaluation waiver from the Ethics Committee of the Ethiopian Public Health Association (Ref: EPHA/OG/2380).
Verbally informed consent was obtained from the participants prior to their participation in the study.

</td>
</tr>
</table>

Powered by Editorial Manager® and ProduXion Manager® from Aries Systems Corporation

**Format for specific study types**<br><br>**Human Subject Research (involving human participants and/or tissue)**<br>• Give the name of the institutional review board or ethics committee that approved the study<br>• Include the approval number and/or a statement indicating approval of this research<br>• Indicate the form of consent obtained (written/oral) or the reason that consent was not obtained (e.g. the data were analyzed anonymously)<br><br>**Animal Research (involving vertebrate animals, embryos or tissues)**<br>• Provide the name of the Institutional Animal Care and Use Committee (IACUC) or other relevant ethics board that reviewed the study protocol, and indicate whether they approved this research or granted a formal waiver of ethical approval<br>• Include an approval number if one was obtained<br>• If the study involved *non-human primates*, add *additional details* about animal welfare and steps taken to ameliorate suffering<br>• If anesthesia, euthanasia, or any kind of animal sacrifice is part of the study, include briefly which substances and/or methods were applied<br><br>**Field Research**<br><br>Include the following details if this study involves the collection of plant, animal, or other materials from a natural setting:<br>• Field permit number<br>• Name of the institution or relevant body that granted permission	
**Data Availability**<br><br>Authors are required to make all data underlying the findings described fully available, without restriction, and from the time of publication. PLOS allows rare exceptions to address legal and ethical concerns. See the PLOS Data Policy and FAQ for detailed information.	Yes - all data are fully available without restriction

A Data Availability Statement describing where the data can be found is required at submission. Your answers to this question constitute the Data Availability Statement and **will be published in the article**, if accepted.<br><br>**Important:** Stating 'data available on request from the author' is not sufficient. If your data are only available upon request, select 'No' for the first question and explain your exceptional situation in the text box.<br><br>Do the authors confirm that all data underlying the findings described in their manuscript are fully available without restriction?	
**Describe where the data may be found in full sentences. If you are copying our sample text, replace any instances of XXX with the appropriate details.**<br><br>• If the data are **held or will be held in a public repository**, include URLs, accession numbers or DOIs. If this information will only be available after acceptance, indicate this by ticking the box below. For example: *All XXX files are available from the XXX database (accession number(s) XXX, XXX.*).<br>• If the data are all contained **within the manuscript and/or Supporting Information files**, enter the following: *All relevant data are within the manuscript and its Supporting Information files.*<br>• If neither of these applies but you are able to provide **details of access elsewhere**, with or without limitations, please do so. For example:<br><br>*Data cannot be shared publicly because of [XXX]. Data are available from the XXX Institutional Data Access / Ethics Committee (contact via XXX) for researchers who meet the criteria for access to confidential data.*<br><br>*The data underlying the results presented in the study are available from (include the name of the third party*	***PA/E AT ACCEPT: Please confirm that DOI provided is functional at accept***<br>The datasets used and/or analyzed during the current study DOI (https://doi.org/10.17037/DATA.00001565) will be available from London School of Hygiene and Tropical Medicine.

and contact information or URL).<br>• This text is appropriate if the data are owned by a third party and authors do not have permission to share the data.<br><br>* typeset	
Additional data availability information:	Tick here if the URLs/accession numbers/DOIs will be available only after acceptance of the manuscript for publication so that we can ensure their inclusion before publication.

Powered by Editorial Manager® and ProduXion Manager® from Aries Systems Corporation

Manuscript Click here to access/download;Manuscript;Manuscript.doc

Key factors influencing motivation among health extension workers and health care professionals in four regions of Ethiopia: a cross-sectional study.

Mehiret Abate*[1]; Zewdie Mulissa[1]; Hema Magge[1,2.] Befikadu Bitewulign[1]; Abiyou Kiflie[1]; Abera Biadgo[1]; Haregeweyni Alemu[1]; Yakob Seman[3]; Dorka Woldesenbet[4]; Abiy Seifu Estifanos[4]; Gareth Parry[5]; Matthew Quaife[6.]

1Institute for Healthcare Improvement, Addis Ababa, Ethiopia

2Division of Global Health Equity, Brigham and Women's Hospital, Boston, MA, United States of America

3Medical Service General Directorate, Ministry of Health of Ethiopia, Addis Ababa, Ethiopia

4Deparment of Reproductive, Family and Population Health, School of Public Health, Addis Ababa University, Addis Ababa, Ethiopia

5Department of Plastic and Oral Surgery, Boston Children's Hospital, 300 Longwood Avenue, Boston, MA 02115, United States of America

6Department of Global Health and Development, London School of Hygiene and Tropical Medicine, London, United Kingdom

*mehiret.ema@gmail.com (MA)

Abstract

Background Although Ethiopia has improved access to health care in recent years, quality of care remains low. Health worker motivation is an important determinant of performance and affects quality of care. Low health care workers motivation can be associated with poor health care quality and client experience, long patient waiting time, non-attendance, and poor clinical outcome. Objective this study sought to determine the extent and variation of health professionals' motivation alongside factors associated with motivation. Methods We conducted a facility based cross-sectional study among health extension workers (HEWs) and health care professionals in four regions: Amhara, Oromia, South nations, and nationalities people's region (SNNPR) and Tigray from April 15 to May 10, 2018. We sampled 401 health system workers: skilled providers including nurses and midwives (n=110), HEWs (n=210); and non-patient facing health system staff representing case team leaders, facility and district heads, directors, and officers (n=81). Participants completed a 30-item Likert scale ranking tool which asked questions across 17 domains. We used exploratory factor analysis to explore latent motivation constructs. Results Of the 397 responses with complete data, 61% (95% CI 56%-66%) self-reported motivation as "very good" or "excellent". Significant variation in motivation was seen across regions with SNNPR scoring significantly lower on a five-point Likert scale by 0.35 points (P=0.003). The exploratory factor analysis identified a three-factors: personal and altruistic goals; pride and personal satisfaction; and recognition and support. The personal and altruistic goals factor varied across regions with Oromia and SNNPR being significantly lower by 0.13 (P=0.018) and 0.12 (P=0.039) Likert points respectively. The pride and personal satisfaction factor were higher among those aged >=30 years by 0.14 Likert scale points (P=0.045) relative to those aged

between 19-24years. Conclusions Overall, motivation was high among participants but varied across region, cadre, and age. Workload, leave, and job satisfaction were associated with motivation.

Introduction

Health worker motivation is an important determinant of health worker performance and, ultimately, health care quality (1,2). Although a range of factors affect health care quality, motivation is an important determinant of health worker effort, retention, and quality of provision, in addition to health care organization, resource availability, and other provider- and patient-related factors (2). Motivation in the work context can be defined as an individual's degree of willingness to exert and maintain an effort towards organizational goals (3,4). Previous research has identified a number of factors that can affect health worker motivation, which can be broadly categorized in to three areas: social factors such as community expectations and social values; organizational factors such as resources and managerial support; and individual or process factors such as preferences or intrinsic attitudes (5,6).

Several comparable studies have been conducted in sub-Saharan Africa. A study in Tanzania indicated that 45% of the individuals working in a primary health care unit were unsatisfied with their job. The reasons cited for dissatisfaction were low salaries, factors related to the working environment, and inadequate facilities for performing expected tasks (7). A systematic review to assess motivation and retention of health care workers in developing countries indicated the importance of financial incentive on health workers motivation (8). The studies indicated that health workers take pride and are motivated when they feel they could progress. Recognition by the employer and community was one of the most motivating factors for health workers alongside education and training opportunities. Low salaries were demotivating factors for health care provider (5,8–10,10–14). Low health care workers motivation is a major contributor to the poor health service quality and client

experience, long waiting time, nonattendance, and unofficial fee charges and poor clinical outcome (15).

Three previous studies have assessed health worker motivation in Ethiopia quantitatively, though all were small in scale and focused on specific health worker cadres (3,9,16). A study conducted in central Ethiopia revealed that the overall motivation of health professionals working in different hospitals of West Shewa Zone was reasonably high. Motivation was affected by financial incentives. The mean motivation score among health professionals who received monthly financial benefit was significantly higher than those who did not. Health professionals who had master's degrees and doctors had highest motivation. Professionals who worked for less than five years had less motivation (3). A study done in public hospitals of West Amhara in Northwest Ethiopia revealed that the mean overall motivation scores were similarly high. Highly trained professionals, young age groups, and professionals who received performance evaluations and professional development promotions were more motivated (16).

However, health care professionals' motivation working in public health centres in Gedeo Zone of South Nations and nationalities people's region (SNNPR) was very low. Lack of recognition and appreciation from their immediate supervisor or manager decreased their motivation. Work experience was a positive predictor of job motivation as work experience increased, job motivation increased (9). A qualitative study indicated that Ethiopian primary health care workers commonly face work-related stress and experience features of burnout, which may contribute to the high turnover of staff and dissatisfaction of both patients and providers (17).

Though research has been done to identify factors associated with motivation in public hospitals and health centres in different regions of Ethiopia, there has been limited research conducted to explore the extent of motivation among Health extension workers (HEWs) working in the community and midlevel health care professionals across the four large regions of Ethiopia, or how motivation differs across cadres and regions. Thus, this study is sought to determine the extent and variation of health professionals' motivation alongside factors associated with motivation. We collected data from the four regions of Ethiopia, home to 81% of the country's population: Amhara; Oromia; SNNPR; and Tigray and accommodates most health facilities and health workers. These regions are highly diverse population and have different geographic characteristics improving representativeness (18).

Materials and Methods

Tool development

We adapted a motivation tool developed and validated among community health workers (CHWs) in Uganda (19), making minor changes to wording to suit the Ethiopian context. The tool consisted of 17 questions. We added 8 additional questions from a health worker motivation evaluation conducted in Tanzania to explore extrinsic motivating factors in more depth (20). Finally, we included 5 further questions relating to activities related to the quality improvement programme being implemented in our sample. The final tool is shown in Table 1.

Table 1. Motivation questions included in survey, source, and domain from April 15 - May 10, 2018

Domain	Wording	Source
Altruism	My work is important because I help people	(19)
Altruism	As long as I can do what I enjoy, I am not that concerned about exactly what income or awards I earn	QI-specific indicator
Community	I am respected in my community for the work I do	(19)
Income	I am strongly motivated by the income I can earn at work	(20)
Income	To be motivating, hard work must be rewarded with more status and money.	(20)
Income	My salary accurately reflects my skills and workload	(20)
Intention to leave	I intend to stop working in this role in the next 12 months	(19)
Job satisfaction	I am proud of the work I do	(20)
Job satisfaction	In general, I am satisfied with my role	(19)
Knowledge gain	I gain knowledge from being in this role	(19)
Knowledge gain	Training sessions that I attend are worthwhile and add benefit to my career path	QI-specific indicator
Motivation	At the moment I do not feel like working as hard as I can	(20)
Motivation	I feel like performing the duties required of me	(19)
Needs satisfaction	I am strongly motivated by the recognition I get from other people	(20)
Needs satisfaction	It is important that I do a good job so that the health system works well	(19)
Needs satisfaction	My job makes me feel good about myself.	(20)
Needs satisfaction	I feel it is not so important doing a good job if nobody else knows about it	QI-specific indicator
Organisational citizenship	I am willing to do more than is asked of me in my role	(19)
Organisational citizenship	Sometimes I do not understand why I am asked to do certain things, but I do them anyway	(19)
Organisational justice	The system of choosing who attends training sessions is fair	QI-specific indicator
Organisational justice	I do not have enough opportunities to attend training sessions to develop my career	QI-specific indicator
Outcome expectancy	I am keenly aware of the career goals I have set for myself	(20)
Outcome expectancy	If I do well at work, I will achieve my goals	(19)
Programme	I am proud to be working in my role	(19)

Programme commitment	I feel committed to my role	(19)
Resource availability	The health system provides everything I need to do my job properly	(19)
Self-efficacy	I can solve most problems I have at work if I work hard	(19)
Supervision	Suggestions made by people like me on how to improve their work are usually ignored by supervisors	(19)
Supervision	My supervisors and managers are supportive of me	(19)
Workload	I can complete all of the work I am expected to do	(19)

All items had Likert scale response options where 1=strongly agree, 2=agree, 3=neutral, 4=disagree, 5=strongly disagree.

Sampling and data collection

We conducted a cross-sectional survey from April 15 to May 10, 2018. The survey was part of a baseline evaluation of a quality improvement (QI) program delivered by the Institute for Healthcare Improvement (IHI) in partnership with the Ministry of Health Ethiopia (MOH). Although the sampling frame of this study is based on the IHI program, data are from pre-intervention baseline data collection, and we do not expect motivation to have been influenced by the intervention at this point. The IHI program was implemented in 19 districts: 7 in Oromia, 5 in Amhara, 5 in SNNPR, and 2 in Tigray. Using a random number generator, we randomly selected one intervention district from each region (Jimma Town, Wogera, Chena, and Degua Tembien respectively). We added one additional randomly selected district in Amhara because Wogera would not have yielded 50 eligible respondents—our target for each region. We further purposively sampled two additional districts from Oromia and SNNPR (Bunno Bedelle and Chencha respectively) where qualitative evaluative work took place, to triangulate findings in a larger evaluation of IHI's QI program. Data collection was conducted by seven research assistants who received one week training at the start of the data collection process and then were matched to their home regions where they have experience working in and speak local language to assist with community entry and mitigate language issues. The data quality was assured by using validated tools, trained data collectors, and conducting interviews in the local languages. The survey was piloted out of 19 district health office staff in December 2017. No changes were made to the survey between piloting and the final survey as it was understood well by participants, assessed through debriefing interviews after survey completion.

In each district, we mapped the hospital, all health centres and health posts, and approached the district health office for permission letters that was later obtained. In each hospital and health centre, we obtained a list of all eligible health care professionals and HEWs. We then randomly selected participants for interviews. In each district, we interviewed around 50 participants across a range of health worker and management cadres, including the heads or clinical directors of the district, each hospital, and each health centre. We interviewed around four maternal and child health care providers from the hospitals and two from each health centre, and around five HEWs from each health centre. A target sample size of 50 respondents per region was chosen, based on the primary research question of assessing changes in motivation as measured by Likert scale questions, in line with a rule of thumb in exploratory factor analysis that 50 participants per cluster is a reasonable sample size to detect differences across clusters (21)

In each hospital or health centre, we obtained a list of all eligible MNH providers and randomly selected participants for interviews. Their names were written in alphabetical order next to a column of randomly generated numbers and interviewers sequentially chose participants from the smallest random number upwards until the requisite number of participants was reached. If participants were not available, we sought to arrange interviews via phone and returned to the facility up to three times before classifying them as unreachable and selecting the next worker from the list. Data were entered on tablet computers using Open Data Kit software (www.opendatakit.org) and exported to STATA V.13.

Data analysis

We categorized the responses according to sociodemographic factors using counts and percentages as appropriate.

To explore the underlying correlations and associations and identify factors within the survey items, we first re-coded the survey items from the 5-point Likert scale from poor to fair, good, very good and excellent to a continuous variable from 1 (poor) to 5 (excellent). Next, we used the re-coded items in an exploratory factor analysis. For the exploratory factor analysis, we first removed items from our list of 30 questions which had poor psychometric performance, removing items which had more than 10% missing data, items which were given the same score of over 80% of participants, and items which did not load on any factors up to a level of 0.4 in initial factor analysis. We used a threshold of 0.4 to assume a strong relationship with a factor, and the optimal number of factors was established through a scree test and multiple runs (22,23). We used maximum likelihood ProMax oblique rotation to reduce the number of variables with high loadings and to allow factors to be correlated. Construct validity was indicated by loading at least three items per factor and absence of substantive cross-loading.

We explored the association of overall motivation and with the motivation factors identified with overall job satisfaction and demographic and structural factors including gender, location, cadre, age, perceived gross salary, work experience, using univariate and multivariate ordinary least squares regression models, and show ordered logistic regression model results in the appendixes 1 & 2. Variables having p value ≤ 0.2 in the bivariate analysis were fitted into a multivariable regression model to control the effects of confounding. Normality assumptions were checked by Schapiro - Francia W tests, and

variance inflation factor estimates were generated for regressors (24). Average job satisfaction was assessed by re-coding the 5-point Likert scale ranging from least satisfied with their job (1) to most satisfied (5) as a continuous variable.

Ethical considerations

Written informed consent was obtained from all participants. The study was undertaken with ethical approval from the Observational Research Ethics Committee of the London School of Hygiene and Tropical Medicine (Ref: 14429) and a program evaluation waiver from the Ethics Committee of the Ethiopian Public Health Association (Ref: EPHA/OG/2380).

Results

Sociodemographic characteristics

Of 401 people surveyed, 397 (99%) responded. Most of the respondents were between 25-30 years of age (232, 58.44%) and female (288, 72.73%). Fifty-one percent of respondents had greater than 4 years of work experience, and 52.39% of the respondents were HEWs shown in Table 2.

Table 2. Background characteristics of participants from four regions, (n=397), April 15 - May 10, 2018

Variables		Number (%)
Region		
	Amhara	81 (20.40)
	Oromia	105 (26.45)
	SNNPR	137 (34.51)
	Tigray	74 (18.64)
Age (Years)		
	19-24	90 (22.67)
	25-30	232 (58.44)
	>30	75 (18.89)
Job title		
	Health extension workers	208 (52.39)
	Care provider (Nurse, Midwife, health officer…) *	109 (27.46)
	Leaders	63 (15.71)
	Other	17 (4.28)
Gender		
	Male	109 (27.46)
	Female	288 (72.54)
Work experience		
	<6month	56 (14.11)
	6month-1year	40 (10.08)
	1-2years	42 (10.58)
	2-4years	54 (13.60)
	>4years	205 (51.64)

Figure 1 plots the responses to each motivational task, where 1 represents “strongly agree” and 5 represents “strongly disagree”, and blue dots represent items where a lower score is a priori better (e.g., “I am respected in my community for the work I do”) and red dots where a lower score is worse (e.g., “I intend to stop working in this role in the next 12 months”).

Figure 1. Plot of responses to motivational survey items among participants from four regions of Ethiopia, April 15 - May10,2018

Motivation construction – Exploratory factor analysis

We ran a factor analysis and ultimately found that the three-factor model fit the data best. We removed 11 items which did not load to 0.4 on any factor. We calculated the final factor score by multiplying the items by the factor score and summing. Table 3 shows the factor loadings. In addition to the factor loading above 0.4, we used eigen values >1 as a criterion to reduce the number of factors into three. We labelled the three factors as personal and altruistic goals; pride and personal satisfaction; and recognition and support. The minimum and maximum score for personal and altruistic goals was 1 and 4.85; for pride and personal satisfaction it was 1 and 4.17; and for recognition and support, it was 1 and 4.5.

Table 3. Exploratory factor analysis results showing the factor loadings by individual items

April 15 - May 10, 2018

**Variable**	**Factor 1: Personal and altruistic goals**	**Factor 2: Pride and personal satisfaction**	**Factor 3: Recognition and support**	**Uniqueness**
My work is important because I help people	0.65			0.44
I am respected in my community for the work I do	0.58			0.51
I am keenly aware of the career goals I have set for myself	0.57			0.50
I feel committed to my role	0.56			0.44
If I do well at work, I will achieve my goals	0.54			0.57
I am willing to do more than is asked of me in my role	0.47			0.68
I can solve most problems I have at work if I work hard	0.37			0.70
I can complete all the work I am expected to do		0.41		0.68
I feel like performing the duties required of me		0.53		0.55
I am proud of the work I do		0.57		0.50
I am proud to be working in my role		0.65		0.43
In general, I am satisfied with my role		0.57		0.64
My job makes me feel good about myself.		0.54		0.57
It is important that I do a good job so that the health system works well			0.48	0.51
Training sessions that I attend are worthwhile and add benefit to my career path			0.49	0.59
To be motivating, hard work must be rewarded with more status and money.			0.58	0.57
I am strongly motivated by the recognition I get from other people			0.44	0.62
I gain knowledge from being in this role			0.62	0.38
My supervisors and managers are supportive of me			0.36	0.73

Legend: Factor 1 interpreted to represent personal and altruistic goals. Factor 2 interpreted to represent pride and personal satisfaction. Factor 3 interpreted to represent recognition and support.

Motivation level

Of the total respondents, 61% were motivated, responding “very good” or “excellent” (95% CI 57% - 66%). The most motivating factor mentioned by 70% of the participants was the opportunity to improve health. The most demotivating factor mentioned by 29% participants was workload (95% CI 34% - 39%).

Factors related to overall motivation

Table 4 shows the results of the regression model with overall motivation as the outcome variable and several explanatory factors fitted.

The overall motivation score mean was 2.18 out of 5 (95% CI 2.10, 2.27; P=0.001). Average job satisfaction was strongly associated with higher motivation (P=0.001). We found variation in motivation across regions, where participants from SNNPR reported lower motivation than the Amhara region by 0.35 (95% CI 0.12, 0.59; P=0.003). Participants who have a medium workload (meaning that they have enough time to complete duties) were also less motivated than participants who have a light workload by 0.48 points (95% CI -0.90, -0.06; P=0.024), though high workload was not significantly different. There was no significant variation in overall motivation with job title and perceived fairness of gross salary.

Table 4. Factors associated with overall motivation among participants form four regions April 15 - May 10, 2018

Factor	Coef.	95% CI	P-value
Region			
Amhara	Reference		
Oromia	0.19	(-0.04, 0.42)	0.110
SNNPR	-0.35	(-0.59, -0.12)	0.003
Tigray	-0.05	(-0.32, 0.21)	0.685
Job title			
HEW	Reference		
Health care providers	0.13	(0.32, -0.05)	0.161
Leaders	-0.19	(0.03, -0.41)	0.096
Other	-0.23	(0.17, -0.64)	0.269
Workload			
Light: more than enough time to complete duties	Reference		
Medium: enough time to complete duties	-0.48	(-0.90, -0.06)	0.024
Heavy: barely enough time to complete duties	-0.29	(0.11, 0.70)	0.163
Perceived gross salary fair			
Very fair	Reference		
Quite fair	0.00	(-0.79, 0.80)	0.995
Neither fair nor unfair	0.29	(-0.54, 1.12)	0.488
Quite unfair	0.10	(-0.69, 0.90)	0.798
Very unfair	-0.04	(-0.87, 0.78)	0.914
Average job satisfaction	0.23	(0.13, 0.34)	0.001

Factor 1: Personal and altruistic goals

The first factor identified items which related to personal success and goal setting and altruism. We refer to this factor as "personal and altruistic goals". The mean response for personal and altruistic goals was 1.61 Likert points (95% CI 1.56, 1.66; P=0.001). From the results of the regression analysis summarized in Table 5, average job satisfaction was strongly associated with higher personal and altruistic goals score (P=0.001). Variation in personal and altruistic goals was seen among regions, where participants from Oromia and SNNPR had lower personal and altruistic goal scores than Amhara region by 0.13 Likert points (95% CI -0.25, -0.02; P=0.018) and 0.12 Likert points (95% CI -0.23, -0.01; P=0.039) respectively. Average leave (days out of work) was another significant factor associated with personal and altruistic goals, where an increase of 1 was associated with a decrease in personal and altruistic goals of 0.08 Likert points (95% CI -0.12, -0.04; P=0.001). Age, gender, and perceived fairness of salary had no significant association with personal and altruistic goals.

Table 5. Association between motivation factors and demographic and structural factors among participants from four regions April 15 - May 10, 2018

	Factor 1: personal and altruistic goals			Factor 2: pride and personal satisfaction			Factor 3: recognition and support		
**Factor**	**Coef.**	**95% CI**	**P-value**	**Coef.**	**95% CI**	**P-value**	**Coef.**	**95% CI**	**P-value**
Gender									
Male	Reference			Reference			Reference		
Female	0.01	(-0.08, 0.09)	0.896	-0.02	(-0.12, 0.08)	0.720	-0.01	(-0.17, 0.14)	0.863
Region									
Amhara	Reference			Reference			Reference		
Oromia	0.13	(-0.02, -0.25)	0.018	0.00	(-0.11, 0.11)	0.969	-0.15	(-0.29, -0.01)	0.034
SNNPR	0.12	(-0.01, -0.23)	0.039	- 0.10	(-0.21, 0.01)	0.064	-0.17	(-0.32, -0.03)	0.017
Tigray	0.04	(-0.17, 0.08)	0.523	0.08	(-0.04, 0.2)	0.175	0.08	(-0.07, 0.24)	0.290
Age									
19-24	Reference			Reference			Reference		
25-30	0.04	(-0.14,0.5)	0.403	0.07	(-0.02, 0.19)	0.180	-0.02	(-0.16, 0.10)	0.710
>30	0.01	(-0.01, 0.13)	0.915	0.14	(0.01, 0.28)	0.045	0.04	(-0.13, 0.21)	0.633
Perceived gross salary fair									
Very fair	Reference						Reference		
Quite fair	-0.27	(0.10, -0.65)	0.155				-0.15	(-0.61, 0.30)	0.514
Neither fair nor unfair	-0.27	(0.12, -0.66)	0.181				0.01	(-0.47, 0.48)	0.976

Quite unfair	-0.21	(0.17, -0.59)	0.277				0.01	(-0.45, 0.46)	0.973
Very unfair	-0.26	(0.13, -0.65)	0.199				0.09	(-0.39, 056)	0.712
Work experience									
<6month				Reference					
6month-1year				-0.02	(-0.18, 0.14)	0.824	-0.35	(-0.83, 0.12)	0.147
1-2years				-0.00	(-0.16, 0.15)	0.971	-0.29	(-0.76, 0.18)	0.227
2-4years				0.11	(-0.37, 0.26)	0.145	-0.34	(-0.80, 0.12)	0.151
>4years				-0.06	(-0.18, 0.07)	0.366	-0.39	(-0.85, 0.07)	0.099
Job title									
HEW							Reference		
Healthcare provides							0.12	(0.00, 0.25)	0.053
Leaders							0.02	(-0.17, 0.20)	0.862
Other							-0.01	(-0.28, 0.25)	0.929
Average job satisfaction	0.34	(0.29, 0.39)	0.001	0.06	(0.09, 0.02)	0.004	0.09	(0.14, 0.04)	0.001
Average leave	-0.08	(-0.04, -0.12)	0.001	0.56	(0.50, 0.61)	0.001	0.35	(0.29, 0.41)	0.001

Factor 2: Pride and personal satisfaction

The second factor consisted of variables relating to the pride and personal satisfaction respondents experienced in their jobs, and the satisfaction their jobs gave them. The mean score of pride and personal satisfaction was 1.78 Likert points (95% CI 1.72, 1.84; P=0.001). A regression analysis in table 5 indicated that pride and personal satisfaction score vary among age groups. There were significantly high scores among those older than 30 years old by 0.14 Likert points (95% CI 0.01, 0.28; P=0.045) as compared to those between the ages of 19-24 years old. Likewise, as average leave (time out of work) increased by 1, pride and personal satisfaction score decreased by 0.06 Likert points (95% CI -0.09, -0.02; P=0.004). Another factor affecting pride and personal satisfaction was average job satisfaction. Average job satisfaction is strongly associated with high pride and personal satisfaction (P=0.001). Gender, Region, and work experience did not have significant association with pride and personal satisfaction.

Factor 3: Recognition and support

Factor 3 synthesised survey items focusing on the recognition and support those participants received from colleagues and seniors. The mean score of recognition and support was 1.77 (95% CI 1.72, 1.83; P=0.001). From the regression analysis indicated in Table 5, factors associated with recognition and support were job title, region, leave (time out of work) and job satisfaction. Recognition and support scores were significantly high among health care providers as compared to HEWs by 0.12 Likert points (95% CI 0.001, 0.25; P=0.053). Compared to Amhara region, recognition and support scores were significantly low among SNNPR by 0.15 Likert points (95% CI -0.32, -0.03; P=0.017) and Oromia region by

0.17 Likert points (95% CI -0.29, -0.01; P=0.034). Another factor affecting recognition and support was job satisfaction. Average job satisfaction is strongly associated with higher recognition and support score (P=0.001). However, as average leave days increased by one, recognition and support scores significantly decreased by 0.09 Likert points (95% CI -0.14, -0.04; P=0.001). Age, work experience, gender, and perception of fairness of salary has no significant association with recognition and support.

Discussion

The extent of overall motivation in respondents across regions and cadres was generally high, but with some variation across regions and other characteristics. The motivation domains identified in a three-factor factor analysis were effort in personal and altruistic goals; pride and personal satisfaction; and recognition (financial and managerial) support.

Our finding of high overall motivation is consistent with several other studies. For example, a study conducted in West Amhara, Northwest Ethiopia showed that 59% of care providers working in hospitals were motivated (16). In West Shewa Zone of Oromia region, the overall mean motivation score of health professionals from three hospitals was 64% (3). In this study, motivation varied significantly from region to region, which previous smaller studies have not been able to explore. This variation is consistent with a study from Kenya that also found motivational factors varied from region to region (11). SNNPR had the lowest motivation, consistent with a study done among health professionals in SNNPR’s Gedeo zone where only 17% of respondents were highly motivated. This could need further exploratory studies (9).

Personal and altruistic goals of health providers were significantly lower among participants from Oromia and SNNPR compared to those from the Amhara region. Average job satisfaction is strongly associated with high efforts in personal and altruistic goal.

Motivation related to pride and personal satisfaction was significantly high among participants older than 30 years of age as compared to those 24 years and younger. This study was consistent with studies from Central Ethiopia and South Ethiopia where age was a major predictor of motivation; as age increased, motivation to do their job increased (9,12). Similar studies in Burkina Faso, Ghana and Tanzania showed that age has a significant effect on motivation (26). Pride and personal satisfaction factor were significantly high among participants whose job satisfaction was high as of different studies in Ethiopia explains (27).

Recognition and support were significantly low among participants from SNNPR and Oromia region and was associated with low motivation. This could be due to instability within the system or the lack of capacity to supervise the health care workers located in a large geographical area. Among participants with increased average leave, recognition support was significantly low. This may explain why participants who were taking leave more were less motivated. Recognition and support were significantly higher among health care providers than among HEWs. Having more recognition (financial and managerial) support was a positive predictor for job satisfaction and motivation (8,28,29).

A strength of this study was that data was collected from four (Amhara, Oromia, SNNPR, Tigray) major regions of Ethiopia where most of the country's population live and the regional variation of motivation was studied at the same time, and the health care workers and health extension workers motivation were compared, in addition this study addressed computing multiple associated factors with the overall motivation and three motivation

factors identified during factor analysis. Nevertheless, limitations remain. Motivation was self-reported and may therefore be subject to acceptability biases in the face-to-face interviews conducted. Knowledge that interviews were conducted by interviewers from a public university and evaluating an IHI programme may have biased responses. The skilled care provider sample was too small to conduct a subgroup analysis among specific groups of interest such as doctors or midwives. Region-level analyses may have been underpowered to variation in motivation, particularly in the presence of heterogeneity in motivation within regions, for example by cadre, or other unobserved variables. The sampling strategy was not optimized to account for or detect potentially important heterogeneity between regions, urban and rural areas, or QI programme areas, which may further reduce power to detect across-region variation. Qualitative work could have been conducted alongside quantitative data collection to understand why variation exists in motivation between regions and personal characteristics. Finally, we were unable to assess the link between health worker motivation and the quality of care provided which remains under-researched.

Conclusion

Motivation was high among the respondents from the four regions. Key factors associated with motivation were region, age, cadre type, workload, job satisfaction, long leave days and work experience. Further studies are needed to explore the reason for variation in motivation across regions and cadre type.

Acknowledgements

We are grateful to all participants for their time in completing various study components. Excellent research assistance in data collection was provided by Teklit Grum, Tsegaab

Temesgen, Zelalem Tedla, Saba Shiferaw, Duresa Endale, Abeba Hailemelekot, Worknesh Daba, Hasna Musema, Selamawit Herpa, Tigist Abera, Samuel Ayele, and Aniley Dagife. The QI program is funded by the Bill and Melinda Gates Foundation and Margaret A. Cargill Philanthropies. We thank our colleagues from Addis Continental Institute of Public Health who provided insight and expertise that greatly assisted with the analysis and write-up of this research. We are especially indebted to Prof. Alemayehu Worku and Dr Walelign Worku for their tireless support in the analysis of the data. We are also grateful to Jane Roessner for her valuable comments on an earlier version of the manuscript and Naomi Fedna for proofreading the final version to fit journal requirement.

Funding

This work was supported by IDEAS—Informed Decisions for Actions to improve maternal and new-born health (http://ideas.lshtm.ac.uk), which is funded through a grant from the Bill & Melinda Gates Foundation to the London School of Hygiene & Tropical Medicine. (Gates Global Health Grant Number: OPP1149259). The funder of this study had no role in the study's design or conduct, data collection, analysis, or interpretation of results, writing of the paper, or decision to submit for publication.

Author's contributions

Conceptualization: Mehiret Abate, Mathew Quaife, Gareth Parry

Data curation: Mehiret Abate, Matthew Quaife

Formal analysis: Mehiret Abate, Matthew Quaife

Investigation: Dorka Woldesenbet, Abiy Seifu Estifanos, Abera Biadgo, Befikadu Bitewulign, Haregeweyni Alemu, Zewdie Mulissa, Mehiret Abate

Methodology: Mehiret Abate, Matthew Quaife, Gareth Parry

Project administration: Mehiret Abate, Hema Magge, Abiyou Kiflie, Abera Biadgo, Befikadu Bitewulign, Haregeweyni Alemu, Mehiret Abate, Zewdie Mulisa

Resources: Hema Magge, Abiyou Kiflie, Matthew Quaife

Supervision: Matthew Quaife, Mehiret Abate, Gareth Parry, Hema Magge,

Writing – original draft: Mehiret Abate.

Writing – review & editing: Mehiret Abate, Yakob Semagn, Abera Biadgo, Befikadu Bitewulign, Haregeweyni Alemu, Zewdie Mulisa, Dorka woldesenbet, Abiy Seifu Estifanos, Abiyou Kiflie, Hema Magge, Gareth Parry, Matthew Quaife

References

1. Franco LM, Bennett S, Kanfer R. Health sector reform and public sector health worker motivation: a conceptual framework. Social Science & Medicine. 2002 Apr;54(8):1255–66.

2. Mosadeghrad AM. Factors Influencing Healthcare Service Quality. International Journal of Health Policy and Management. 2014;3(2):77–89.

3. Dagne T, Beyene W, Berhanu N. Motivation and Factors Affecting It among Health Professionals in the Public Hospitals, Central Ethiopia. Ethiopian Journal of Health Sciences. 2015 Jul 27;25(3):231.

4. Toode K, Routasalo P, Suominen T. Work motivation of nurses: A literature review. International Journal of Nursing Studies. 2011 Feb 1;48(2):246–57.

5. Franco LM, Bennett S, Kanfer R, Stubblebine P. Determinants and consequences of health worker motivation in hospitals in Jordan and Georgia. Soc Sci Med. 2004 Jan;58(2):343–55.

6. Kumar P, Mehra A, Inder D, Sharma N. Organizational commitment and intrinsic motivation of regular and contractual primary health care providers. J Family Med Prim Care. 2016 Mar;5(1):94–100.

7. Mbilinyi, D., Daniel, M.L. & Lie, G.T. Health worker motivation in the context of HIV care and treatment challenges in Mbeya Region, Tanzania: A qualitative study. *BMC Health Serv Res* **11,** 266 (2011).https://doi.org/10.1186/1472-6963-11-266

8. Willis-Shattuck M, Bidwell P, Thomas S, Wyness L, Blaauw D, Ditlopo P. Motivation and retention of health workers in developing countries: a systematic review. BMC Health Serv Res. 2008 Dec 4;8:247.

9. Berhanu N, Ereso BM, Nebeb GT. Job Motivation and Associated Factors Among Health Care Professionals Working in Public Health Facilities of Gedeo Zone, Southern Ethiopia. Clinical Medicine Research. 2017 Feb 28;6(1):9.

10. Negussie N. Relationship Between Rewards and Nurses' Work Motivation in Addis Ababa Hospitals. Ethiop J Health Sci. 2012 Jul;22(2):107–12.

11. Ojakaa D, Olango S, Jarvis J. Factors affecting motivation and retention of primary health care workers in three disparate regions in Kenya. Human Resources for Health. 2014 Jun 6;12(1):33.

12. Beyene W. Work Motivation and Factors affecting it among Health Professionals in West Shoa Zone Public Hospitals, Oromia Region, Central Ethiopia. Ethiop J Health Sci. 2015 Jul 1;25:231–42.

13. Mbindyo, P., Gilson, L., Blaauw, D. *et al.* Contextual influences on health worker motivation in district hospitals in Kenya. *Implementation Sci* **4,** 43 (2009).https://doi.org/10.1186/1748-5908-4-43

14. Lunenburg FC. Self-Efficacy in the Workplace: Implications for Motivation and Performance. International journal of Management, Business and administration.2011;14(1):1-6.

15. Alhassan RK, Spieker N, van Ostenberg P, Ogink A, Nketiah-Amponsah E, de Wit TFR. Association between health worker motivation and healthcare quality efforts in Ghana. Human Resources for Health. 2013 Aug 14;11(1):37.

16. Weldegebriel Z, Ejigu Y, Weldegebreal F, Woldie M. Motivation of health workers and associated factors in public hospitals of West Amhara, Northwest Ethiopia. *Patient Prefer Adherence*. 2016;10:159-169. Published 2016 Feb 15. doi:10.2147/PPA.S90323

17. Selamu, M., Thornicroft, G., Fekadu, A. *et al.* Conceptualisation of job-related wellbeing, stress and burnout among healthcare workers in rural Ethiopia: a qualitative study. *BMC Health Serv Res* **17,** 412 (2017).https://doi.org/10.1186/s12913-017-2370-5

18. Biadgo A, Legesse A, Estifanos AS, Singh K, Mulissa Z, Kiflie A, et al. Quality of maternal and newborn health care in Ethiopia: a cross-sectional study. BMC Health Serv Res. 2021 Dec;21(1):679.

19. Strachan DL, Källander K, Nakirunda M, Ndima S, Muiambo A, Hill Z, et al. Using theory and formative research to design interventions to improve community health worker motivation, retention and performance in Mozambique and Uganda. Hum Resour Health. 2015 Apr 30;13:25.

20. Chandler CIR, Chonya S, Mtei F, Reyburn H, Whitty CJM. Motivation, money and respect: A mixed-method study of Tanzanian non-physician clinicians. Social Science & Medicine. 2009 Jun 1;68(11):2078–88.

21. Borghi J, Lohmann J, Dale E, Meheus F, Goudge J, Oboirien K, et al. How to do (or not to do)… Measuring health worker motivation in surveys in low- and middle-income countries. Health Policy Plan. 2018 Mar;33(2):192–203.

22. Ferguson, E. and T. Cox,. Exploratory factor analysis: A users' guide. International journal of selection and assessment. 1993;1(2):84–94.

23. Hilari, K. et. al. Stroke and aphasia quality of life scale-39 (SAQOL-39) evaluation of acceptability, reliability, and validity. Stroke, . 2003;34(8):1944–50.

24. Akinwande, M.O., Dikko, H.G. and Samson, A. (2015) Variance Inflation Factor: As a Condition for the Inclusion of Suppressor Variable(s) in Regression Analysis. Open Journal of Statistics, 5, 754-767.

26. Prytherch H, Leshabari MT, Wiskow C, Aninanya GA, Kakoko DCV, Kagoné M, et al. The challenges of developing an instrument to assess health provider motivation at primary

care level in rural Burkina Faso, Ghana and Tanzania. Global Health Action. 2012 Dec;5(1):19120.

27. Hotchkiss DR, Banteyerga H, Tharaney M. Job satisfaction and motivation among public sector health workers: evidence from Ethiopia. Hum Resour Health. 2015 Dec;13(1):83.

28. Daneshkohan A, Zarei E, Mansouri T, Maajani K, Ghasemi MS, Rezaeian M. Factors Affecting Job Motivation among Health Workers: A Study From Iran. Global Journal of Health Science. 2015;7(3):8.

29. Deschamps C, Rinfret N, Lagacé MC, Privé C. Transformational Leadership and Change: How Leaders Influence Their Followers' Motivation Through Organizational Justice. J Healthc Manag. 2016 Jun;61(3):194–213.

Supporting information

S1 Questionnaire. Questionnaire used for data collection in ODK file was prepared in both local (Amharic) and English language.

S2 Appendix 1. Table A1. Results from ordered logit model showing factors associated with overall motivation among participants form four regions April 15- May10, 2018

S3 Appendix 2. Table A2. Results from ordered logit model showing association between motivation factors and demographic and structural factors among participants from four regions April 15- May10,2018

S4 The datasets used and/or analysed during the current study DOI (https://doi.org/10.17037/DATA.00001565) will be available from London School of Hygiene and Tropical Medicine.

a plot of responses to motivational survey items
Click here to access/download;Figure;Fig1.tif.tif

Figure 1. Plot of responses to motivational survey items among participants from four regions of Ethiopia, April 15 - May 10, 2018.

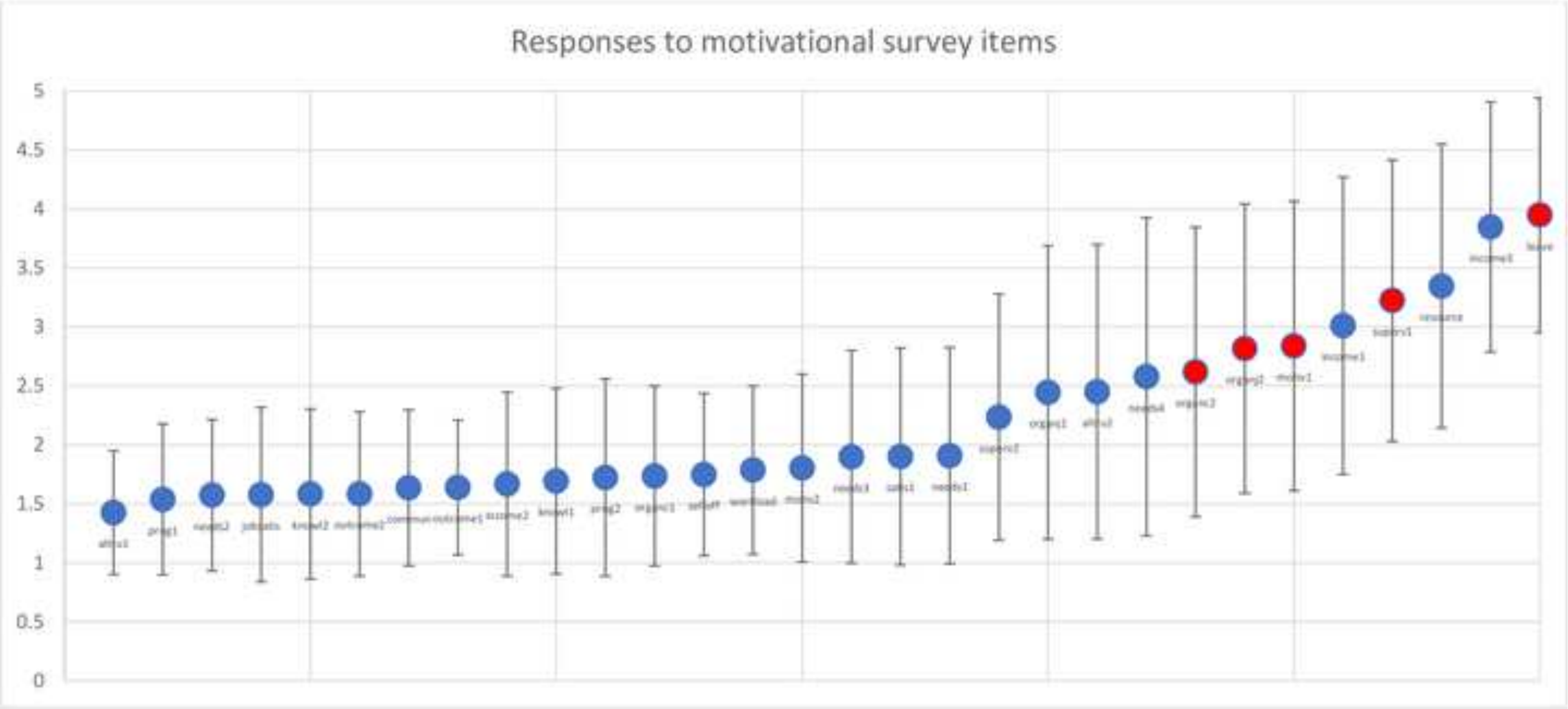

Questionnaire used for data collection in Amharic and English languages

Click here to access/download
Supporting Information
S1_Questionnaire. ODK.xlsx

Appendix1 table A1 results from ordered logit model showing factors associated with overall motivation

Click here to access/download
Supporting Information
S2_Appendix1.pdf.pdf

Appendix2 table A2 results from ordered logit model showing association between motivation factors and demographic

Click here to access/download

Supporting Information

S3_Appendix2 .pdf.pdf

Key factors influencing motivation among health extension workers and health care professionals in four regions of Ethiopia: a cross-sectional study.

Mehiret Abate*[1]; Zewdie Mulissa[1]; Hema Magge[1,2.] Befikadu Bitewulign[1]; Abiyou Kiflie[1]; Abera Biadgo[1]; Haregeweyni Alemu[1]; Yakob Seman[3]; Dorka Woldesenbet[4]; Abiy Seifu Estifanos[4]; Gareth Parry[5]; Matthew Quaife[6.]

1Institute for Healthcare Improvement, Addis Ababa, Ethiopia

2Division of Global Health Equity, Brigham and Women's Hospital, Boston, MA, United States of America

3Medical Service General Directorate, Ministry of Health of Ethiopia, Addis Ababa, Ethiopia

4Deparment of Reproductive, Family and Population Health, School of Public Health, Addis Ababa University, Addis Ababa, Ethiopia

5Department of Plastic and Oral Surgery, Boston Children's Hospital, 300 Longwood Avenue, Boston, MA 02115, United States of America

6Department of Global Health and Development, London School of Hygiene and Tropical Medicine, London, United Kingdom

*mehiret.ema@gmail.com (MA)

Abstract

Background Although Ethiopia has improved access to health care in recent years, quality of care remains low. Health worker motivation is an important determinant of performance and affects quality of care. Low health care workers motivation can be associated with poor health care quality and client experience, long patient waiting time, non-attendance, and poor clinical outcome. Objective this study sought to determine the extent and variation of health professionals' motivation alongside factors associated with motivation. Methods We conducted a facility based cross-sectional study among health extension workers (HEWs) and health care professionals in four regions: Amhara, Oromia, South nations, and nationalities people's region (SNNPR) and Tigray from April 15 to May 10, 2018. We sampled 401 health system workers: skilled providers including nurses and midwives (n=110), HEWs (n=210); and non-patient facing health system staff representing case team leaders, facility and district heads, directors, and officers (n=81). Participants completed a 30-item Likert scale ranking tool which asked questions across 17 domains. We used exploratory factor analysis to explore latent motivation constructs. Results Of the 397 responses with complete data, 61% (95% CI 56%-66%) self-reported motivation as "very good" or "excellent". Significant variation in motivation was seen across regions with SNNPR scoring significantly lower on a five-point Likert scale by 0.35 points (P=0.003). The exploratory factor analysis identified a three-factors: personal and altruistic goals; pride and personal satisfaction; and recognition and support. The personal and altruistic goals factor varied across regions with Oromia and SNNPR being significantly lower by 0.13 (P=0.018) and 0.12 (P=0.039) Likert points respectively. The pride and personal satisfaction factor were higher among those aged >=30 years by 0.14 Likert scale points (P=0.045) relative to those aged

Formatted: Highlight

Formatted: Font: (Default) Calibri, Highlight

Formatted: Font: (Default) Calibri, Highlight

Formatted: Font: (Default) Calibri, Highlight

between 19-24years. Conclusions Overall, motivation was high among participants but varied across region, cadre, and age. Workload, leave, and job satisfaction were associated with motivation.

Introduction

Health worker motivation is an important determinant of health worker performance and, ultimately, health care quality (1,2). Although a range of factors affect health care quality, motivation is an important determinant of health worker effort, retention, and quality of provision, in addition to health care organization, resource availability, and other provider- and patient-related factors (2). Motivation in the work context can be defined as an individual's degree of willingness to exert and maintain an effort towards organizational goals (3,4). Previous research has identified a number of factors that can affect health worker motivation, which can be broadly categorized in to three areas: social factors such as community expectations and social values; organizational factors such as resources and managerial support; and individual or process factors such as preferences or intrinsic attitudes (5,6).

Several comparable studies have been conducted in sub-Saharan Africa. A study in Tanzania indicated that 45% of the individuals working in a primary health care unit were unsatisfied with their job. The reasons cited for dissatisfaction were low salaries, factors related to the working environment, and inadequate facilities for performing expected tasks (7). A systematic review to assess motivation and retention of health care workers in developing countries indicated the importance of financial incentive on health workers motivation (8). The studies indicated that health workers take pride and are motivated when they feel they could progress. Recognition by the employer and community was one of the most motivating factors for health workers alongside education and training opportunities. Low salaries were demotivating factors for health care provider (5,8–10,10–14). Low health care workers motivation is a major contributor to the poor health service quality and client

Formatted: Font: (Default) Calibri, Highlight

Formatted: Highlight

Formatted: Font: (Default) Calibri, Highlight

experience, long waiting time, nonattendance, and unofficial fee charges and poor clinical outcome (15).

Three previous studies have assessed health worker motivation in Ethiopia quantitatively, though all were small in scale and focused on specific health worker cadres (3,9,16). A study conducted in central Ethiopia revealed that the overall motivation of health professionals working in different hospitals of West Shewa Zone was reasonably high. Motivation was affected by financial incentives. The mean motivation score among health professionals who received monthly financial benefit was significantly higher than those who did not. Health professionals who had master's degrees and doctors had highest motivation. Professionals who worked for less than five years had less motivation (3). A study done in public hospitals of West Amhara in Northwest Ethiopia revealed that the mean overall motivation scores were similarly high. Highly trained professionals, young age groups, and professionals who received performance evaluations and professional development promotions were more motivated (16).

However, health care professionals' motivation working in public health centres in Gedeo Zone of South Nations and nationalities people's region (SNNPR) was very low. Lack of recognition and appreciation from their immediate supervisor or manager decreased their motivation. Work experience was a positive predictor of job motivation as work experience increased, job motivation increased (9). A qualitative study indicated that Ethiopian primary health care workers commonly face work-related stress and experience features of burnout, which may contribute to the high turnover of staff and dissatisfaction of both patients and providers (17).

Formatted: Font: (Default) Calibri

Field Code Changed

Though research has been done to identify factors associated with motivation in public hospitals and health centres in different regions of Ethiopia, there has been limited research conducted to explore the extent of motivation among Health extension workers (HEWs) working in the community and midlevel health care professionals across the four large regions of Ethiopia, or how motivation differs across cadres and regions. Thus, this study is sought to determine the extent and variation of health professionals' motivation alongside factors associated with motivation. We collected data from the four regions of Ethiopia, home to 81% of the country's population: Amhara; Oromia; SNNPR; and Tigray and accommodates most health facilities and health workers. These regions are highly diverse population and have different geographic characteristics improving representativeness (18).

Materials and Methods

Tool development

We adapted a motivation tool developed and validated among community health workers (CHWs) in Uganda (19), making minor changes to wording to suit the Ethiopian context. The tool consisted of 17 questions. We added 8 additional questions from a health worker motivation evaluation conducted in Tanzania to explore extrinsic motivating factors in more depth (20). Finally, we included 5 further questions relating to activities related to the quality improvement programme being implemented in our sample. The final tool is shown in Table 1.

Formatted: Highlight

Formatted: Highlight

Table 1. Motivation questions included in survey, source, and domain from April 15 - May 10, 2018

Domain	Wording	Source
Altruism	My work is important because I help people	(19)
Altruism	As long as I can do what I enjoy, I am not that concerned about exactly what income or awards I earn	QI-specific indicator
Community	I am respected in my community for the work I do	(19)
Income	I am strongly motivated by the income I can earn at work	(20)
Income	To be motivating, hard work must be rewarded with more status and money.	(20)
Income	My salary accurately reflects my skills and workload	(20)
Intention to leave	I intend to stop working in this role in the next 12 months	(19)
Job satisfaction	I am proud of the work I do	(20)
Job satisfaction	In general, I am satisfied with my role	(19)
Knowledge gain	I gain knowledge from being in this role	(19)
Knowledge gain	Training sessions that I attend are worthwhile and add benefit to my career path	QI-specific indicator
Motivation	At the moment I do not feel like working as hard as I can	(20)
Motivation	I feel like performing the duties required of me	(19)
Needs satisfaction	I am strongly motivated by the recognition I get from other people	(20)
Needs satisfaction	It is important that I do a good job so that the health system works well	(19)
Needs satisfaction	My job makes me feel good about myself.	(20)
Needs satisfaction	I feel it is not so important doing a good job if nobody else knows about it	QI-specific indicator
Organisational citizenship	I am willing to do more than is asked of me in my role	(19)
Organisational citizenship	Sometimes I do not understand why I am asked to do certain things, but I do them anyway	(19)
Organisational justice	The system of choosing who attends training sessions is fair	QI-specific indicator
Organisational justice	I do not have enough opportunities to attend training sessions to develop my career	QI-specific indicator
Outcome expectancy	I am keenly aware of the career goals I have set for myself	(20)
Outcome expectancy	If I do well at work, I will achieve my goals	(19)
Programme	I am proud to be working in my role	(19)

Programme commitment	I feel committed to my role	(19)
Resource availability	The health system provides everything I need to do my job properly	(19)
Self-efficacy	I can solve most problems I have at work if I work hard	(19)
Supervision	Suggestions made by people like me on how to improve their work are usually ignored by supervisors	(19)
Supervision	My supervisors and managers are supportive of me	(19)
Workload	I can complete all of the work I am expected to do	(19)

All items had Likert scale response options where 1=strongly agree, 2=agree, 3=neutral, 4=disagree, 5=strongly disagree.

Sampling and data collection

We conducted a cross-sectional survey from April 15 to May 10, 2018. The survey was part of a baseline evaluation of a quality improvement (QI) program delivered by the Institute for Healthcare Improvement (IHI) in partnership with the Ministry of Health Ethiopia (MOH). Although the sampling frame of this study is based on the IHI program, data are from pre-intervention baseline data collection, and we do not expect motivation to have been influenced by the intervention at this point. The IHI program was implemented in 19 districts: 7 in Oromia, 5 in Amhara, 5 in SNNPR, and 2 in Tigray. Using a random number generator, we randomly selected one intervention district from each region (Jimma Town, Wogera, Chena, and Degua Tembien respectively). We added one additional randomly selected district in Amhara because Wogera would not have yielded 50 eligible respondents—our target for each region. We further purposively sampled two additional districts from Oromia and SNNPR (Bunno Bedelle and Chencha respectively) where qualitative evaluative work took place, to triangulate findings in a larger evaluation of IHI's QI program. Data collection was conducted by seven research assistants who received one week training at the start of the data collection process and then were matched to their home regions where they have experience working in and speak local language to assist with community entry and mitigate language issues. The data quality was assured by using validated tools, trained data collectors, and conducting interviews in the local languages. The survey was piloted out of 19 district health office staff in December 2017. No changes were made to the survey between piloting and the final survey as it was understood well by participants, assessed through debriefing interviews after survey completion.

In each district, we mapped the hospital, all health centres and health posts, and approached the district health office for permission letters that was later obtained. In each hospital and health centre, we obtained a list of all eligible health care professionals and HEWs. We then randomly selected participants for interviews. In each district, we interviewed around 50 participants across a range of health worker and management cadres, including the heads or clinical directors of the district, each hospital, and each health centre. We interviewed around four maternal and child health care providers from the hospitals and two from each health centre, and around five HEWs from each health centre. A target sample size of 50 respondents per region was chosen, based on the primary research question of assessing changes in motivation as measured by Likert scale questions, in line with a rule of thumb in exploratory factor analysis that 50 participants per cluster is a reasonable sample size to detect differences across clusters (21)

In each hospital or health centre, we obtained a list of all eligible MNH providers and randomly selected participants for interviews. Their names were written in alphabetical order next to a column of randomly generated numbers and interviewers sequentially chose participants from the smallest random number upwards until the requisite number of participants was reached. If participants were not available, we sought to arrange interviews via phone and returned to the facility up to three times before classifying them as unreachable and selecting the next worker from the list. Data were entered on tablet computers using Open Data Kit software (www.opendatakit.org) and exported to STATA V.13.

Data analysis

We categorized the responses according to sociodemographic factors using counts and percentages as appropriate.

To explore the underlying correlations and associations and identify factors within the survey items, we first re-coded the survey items from the 5-point Likert scale from poor to fair, good, very good and excellent to a continuous variable from 1 (poor) to 5 (excellent). Next, we used the re-coded items in an exploratory factor analysis. For the exploratory factor analysis, we first removed items from our list of 30 questions which had poor psychometric performance, removing items which had more than 10% missing data, items which were given the same score of over 80% of participants, and items which did not load on any factors up to a level of 0.4 in initial factor analysis. We used a threshold of 0.4 to assume a strong relationship with a factor, and the optimal number of factors was established through a scree test and multiple runs (22,23). We used maximum likelihood ProMax oblique rotation to reduce the number of variables with high loadings and to allow factors to be correlated. Construct validity was indicated by loading at least three items per factor and absence of substantive cross-loading.

We explored the association of overall motivation and with the motivation factors identified with overall job satisfaction and demographic and structural factors including gender, location, cadre, age, perceived gross salary, work experience, using univariate and multivariate ordinary least squares regression models, and show ordered logistic regression model results in the appendixes 1 & 2. Variables having p value ≤ 0.2 in the bivariate analysis were fitted into a multivariable regression model to control the effects of confounding. Normality assumptions were checked by Schapiro - Francia W tests, and

variance inflation factor estimates were generated for regressors (24). Average job satisfaction was assessed by re-coding the 5-point Likert scale ranging from least satisfied with their job (1) to most satisfied (5) as a continuous variable.

Formatted: Highlight

Ethical considerations

Written informed consent was obtained from all participants. The study was undertaken with ethical approval from the Observational Research Ethics Committee of the London School of Hygiene and Tropical Medicine (Ref: 14429) and a program evaluation waiver from the Ethics Committee of the Ethiopian Public Health Association (Ref: EPHA/OG/2380).

Results

Sociodemographic characteristics

Of 401 people surveyed, 397 (99%) responded. Most of the respondents were between 25-30 years of age (232, 58.44%) and female (288, 72.73%). Fifty-one percent of respondents had greater than 4 years of work experience, and 52.39% of the respondents were HEWs shown in Table 2.

Table 2. Background characteristics of participants from four regions, (n=397), April 15 - May 10, 2018

Variables		Number (%)
Region		
	Amhara	81 (20.40.93)
	Oromia	105 (26.45)
	SNNPR	137 (34.51)
	Tigray	74 (18.64)
Age (Years)		
	19-24	90 (22.67)
	25-30	232 (58.44)
	>30	75 (18.89)
Job title		
	Health extension workers	208 (52.39)
	Care provider (Nurse, Midwife, health officer…) *	109 (27.46)
	Leaders	63 (15.71)
	Other	17 (4.28)
Gender		
	Male	109 (27.46)
	Female	288 (72.54)
Work experience		
	<6month	56 (14.11)
	6month-1year	40 (10.08)
	1-2years	42 (10.58)
	2-4years	54 (13.60)
	>4years	205 (51.64)

Figure 1 plots the responses to each motivational task, where 1 represents “strongly agree” and 5 represents “strongly disagree”, and blue dots represent items where a lower score is a priori better (e.g., “I am respected in my community for the work I do”) and red dots where a lower score is worse (e.g., “I intend to stop working in this role in the next 12 months”).

Figure 1. Plot of responses to motivational survey items among participants from four regions of Ethiopia, April 15 - May10,2018

Motivation construction – Exploratory factor analysis

We ran a factor analysis and ultimately found that the three-factor model fit the data best. We removed 11 items which did not load to 0.4 on any factor. We calculated the final factor score by multiplying the items by the factor score and summing. Table 3 shows the factor loadings. In addition to the factor loading above 0.4, we used eigen values >1 as a criterion to reduce the number of factors into three. We labelled the three factors as personal and altruistic goals; pride and personal satisfaction; and recognition and support. The minimum and maximum score for personal and altruistic goals was 1 and 4.85; for pride and personal satisfaction it was 1 and 4.17; and for recognition and support, it was 1 and 4.5.

Table 3. Exploratory factor analysis results showing the factor loadings by individual items April 15 - May 10, 2018

Variable	Factor 1: Personal and altruistic goals	Factor 2: Pride and personal satisfaction	Factor 3: Recognition and support	Uniqueness
My work is important because I help people	0.65			0.44
I am respected in my community for the work I do	0.58			0.51
I am keenly aware of the career goals I have set for myself	0.57			0.50
I feel committed to my role	0.56			0.44
If I do well at work, I will achieve my goals	0.54			0.57
I am willing to do more than is asked of me in my role	0.47			0.68
I can solve most problems I have at work if I work hard	0.37			0.70
I can complete all the work I am expected to do		0.41		0.68
I feel like performing the duties required of me		0.53		0.55
I am proud of the work I do		0.57		0.50
I am proud to be working in my role		0.65		0.43
In general, I am satisfied with my role		0.57		0.64
My job makes me feel good about myself.		0.54		0.57
It is important that I do a good job so that the health system works well			0.48	0.51
Training sessions that I attend are worthwhile and add benefit to my career path			0.49	0.59
To be motivating, hard work must be rewarded with more status and money.			0.58	0.57
I am strongly motivated by the recognition I get from other people			0.44	0.62
I gain knowledge from being in this role			0.62	0.38
My supervisors and managers are supportive of me			0.36	0.73

Legend: Factor 1 interpreted to represent personal and altruistic goals. Factor 2 interpreted to represent pride and personal satisfaction. Factor 3 interpreted to represent recognition and support.

Motivation level

Of the total respondents, 61% were motivated, responding “very good” or “excellent” (95% CI 57% - 66%). The most motivating factor mentioned by 70% of the participants was the opportunity to improve health. The most demotivating factor mentioned by 29% participants was workload (95% CI 34% - 39%).

Factors related to overall motivation

Table 4 shows the results of the regression model with overall motivation as the outcome variable and several explanatory factors fitted.

The overall motivation score mean was 2.18 out of 5 (95% CI 2.10, 2.27; P=0.001). Average job satisfaction was strongly associated with higher motivation (P=0.001). We found variation in motivation across regions, where participants from SNNPR reported lower motivation than the Amhara region by 0.35 (95% CI 0.12, 0.59; P=0.003). Participants who have a medium workload (meaning that they have enough time to complete duties) were also less motivated than participants who have a light workload by 0.48 points (95% CI -0.90, -0.06; P=0.024), though high workload was not significantly different. There was no significant variation in overall motivation with job title and perceived fairness of gross salary.

Formatted: Highlight

Formatted: Highlight

Formatted: Highlight

Table 4. Factors associated with overall motivation among participants form four regions April 15 - May 10, 2018

Factor	Coef.	95% CI	P-value
Region			
Amhara	Reference		
Oromia	0.19	(-0.04, 0.42)	0.110
SNNPR	-0.35	(-0.59, -0.12)	0.003
Tigray	-0.05	(-0.32, 0.21)	0.685
Job title			
HEW	Reference		
Health care providers	0.13	(0.32, -0.05)	0.161
Leaders	-0.19	(0.03, -0.41)	0.096
Other	-0.23	(0.17, -0.64)	0.269
Workload			
Light: more than enough time to complete duties	Reference		
Medium: enough time to complete duties	-0.48	(-0.90, -0.06)	0.024
Heavy: barely enough time to complete duties	-0.29	(0.11, 0.70)	0.163
Perceived gross salary fair			
Very fair	Reference		
Quite fair	0.00	(-0.79, 0.80)	0.995
Neither fair nor unfair	0.29	(-0.54, 1.12)	0.488~~9~~
Quite unfair	0.10	(-0.69, 0.90)	0.798~~80~~
Very unfair	-0.04	(-0.87, 0.78)	0.914
Average job satisfaction	0.23	(0.13, 0.34)	0.001

Formatted: Highlight

Formatted: Highlight

Formatted: Highlight

Formatted: Highlight

Factor 1: Personal and altruistic goals

The first factor identified items which related to personal success and goal setting and altruism. We refer to this factor as "personal and altruistic goals". The mean response for personal and altruistic goals was 1.61 Likert points (95% CI 1.56, 1.66~~7~~; P=0.001). From the results of the regression analysis summarized in Table 5, average job satisfaction was strongly associated with higher personal and altruistic goals score (P=0.001). Variation in personal and altruistic goals was seen among regions, where participants from Oromia and SNNPR had lower personal and altruistic goal scores than Amhara region by 0.13 Likert points (95% CI -0.25, -0.02; P=0.018) and 0.12 Likert points (95% CI -0.23, -0.01; P=0.039) respectively. Average leave (days out of work) was another significant factor associated with personal and altruistic goals, where an increase of 1 was associated with a decrease in personal and altruistic goals of 0.08 Likert points (95% CI -0.12, -0.04; P=0.001). Age, gender, and perceived fairness of salary had no significant association with personal and altruistic goals.

Formatted: Highlight

Formatted: Highlight

Formatted: Highlight

Formatted: Highlight

Table 5. Association between motivation factors and demographic and structural factors among participants from four regions April 15 - May 10, 2018

	Factor 1: personal and altruistic goals			Factor 2: pride and personal satisfaction			Factor 3: recognition and support		
**Factor**	**Coef.**	**95% CI**	**P-value**	**Coef.**	**95% CI**	**P-value**	**Coef.**	**95% CI**	**P-value**
Gender									
Male	Reference			Reference			Reference		
Female	0.01	(-0.08, 0.09)	0.896	-0.02~~18~~	(-0.12, 0.08)	0.720	-0.01	(-0.17, 0.14)	0.863
Region									
Amhara	Reference			Reference			Reference		
Oromia	0.13	(-0.02, -0.25)	0.018	0.00~~2~~	(-0.11, 0.11)	0.969	-0.15	(-0.29, -0.01)	0.034
SNNPR	0.12	(-0.01, -0.23)	0.039	- 0.10~~4~~	(-0.21, 0.01~~06~~)	0.064	-0.17	(-0.32, -0.03)	0.017
Tigray	0.04	(-0.17, 0.08)	0.523	0.08	(-0.04, 0.2)	0.175	0.08	(-0.07, 0.24)	0.290
Age									
19-24	Reference			Reference			Reference		
25-30	0.04	(-0.14,0.5)	0.403	0.07	(-0.02, 0.19)	0.180	-0.02	(-0.16, 0.10)	0.710
>30	0.01	(-0.01~~2~~, 0.13)	0.915	0.14	(0.01, 0.28)	0.045	0.04	(-0.13, 0.21)	0.633
Perceived gross salary fair									
Very fair	Reference						Reference		
Quite fair	-0.27	(0.10, -0.65)	0.155				-0.15	(-0.61, 0.30)	0.514
Neither fair nor unfair	-0.27	(0.12, -0.66)	0.181				0.01	(-0.47, 0.48)	0.976

Formatted: Highlight

Formatted: Highlight

Formatted: Highlight

Formatted: Highlight

Formatted: Highlight

Quite unfair	-0.21	(0.17, -0.59)	0.277				0.01	(-0.45, 0.46)	0.973
Very unfair	-0.26	(0.13, -0.65)	0.199				0.09	(-0.39, 056)	0.712
Work experience									
<6month				Reference					
6month-1year				-0.02~~18~~	(-0.18, 0.14)	0.824	-0.35	(-0.83, 0.12)	0.147
1-2years				-0.00~~3~~	(-0.16, 0.15)	0.971	-0.29	(-0.76, 0.18)	0.227
2-4years				0.11~~09~~	(-0.37, 0.26)	0.145	-0.34	(-0.80, 0.12)	0.151
>4years				-0.06~~58~~	(-0.18, 0.07)	0.366	-0.39	(-0.85, 0.07)	0.099
Job title									
HEW							Reference		
Healthcare provides							0.12	(0.00~~1~~, 0.25)	0.053
Leaders							0.02	(-0.17, 0.20)	0.862
Other							-0.01	(-0.28, 0.25)	0.929
Average job satisfaction	0.34	(0.29, 0.39)	0.001	0.06	(0.09, 0.02)	0.004	0.09	(0.14, 0.04)	0.001
Average leave	-0.08	(-0.04, -0.12)	0.001	0.56	(0.50, 0.61)	0.001	0.35	(0.29, 0.41)	0.001

Formatted: Highlight

Formatted: Highlight

Formatted: Highlight

Formatted: Highlight

Formatted: Highlight

Factor 2: Pride and personal satisfaction

The second factor consisted of variables relating to the pride and personal satisfaction respondents experienced in their jobs, and the satisfaction their jobs gave them. The mean score of pride and personal satisfaction was 1.78 Likert points (95% CI 1.72, 1.84; P=0.001). A regression analysis in table 5 indicated that pride and personal satisfaction score vary among age groups. There were significantly high scores among those older than 30 years old by 0.14 Likert points (95% CI 0.01, 0.28; P=0.045) as compared to those between the ages of 19-24 years old. Likewise, as average leave (time out of work) increased by 1, pride and personal satisfaction score decreased by 0.06 Likert points (95% CI -0.09, -0.02; P=0.004). Another factor affecting pride and personal satisfaction was average job satisfaction. Average job satisfaction is strongly associated with high pride and personal satisfaction (P=0.001). Gender, Region, and work experience did not have significant association with pride and personal satisfaction.

Formatted: Highlight

Formatted: Highlight

Formatted: Highlight

Formatted: Highlight

Factor 3: Recognition and support

Factor 3 synthesised survey items focusing on the recognition and support those participants received from colleagues and seniors. The mean score of recognition and support was 1.77 (95% CI 1.72, 1.83; P=0.001). From the regression analysis indicated in Table 5, factors associated with recognition and support were job title, region, leave (time out of work) and job satisfaction. Recognition and support scores were significantly high among health care providers as compared to HEWs by 0.12 Likert points (95% CI 0.001, 0.25; P=0.053). Compared to Amhara region, recognition and support scores were significantly low among SNNPR by 0.15 Likert points (95% CI -0.32, -0.03; P=0.017) and Oromia region by

Formatted: Highlight

Formatted: Highlight

Formatted: Highlight

Formatted: Highlight

Formatted: Highlight

0.17 Likert points (95% CI -0.29, -0.01; P=0.034). Another factor affecting recognition and support was job satisfaction. Average job satisfaction is strongly associated with higher recognition and support score (P=0.001). However, as average leave days increased by one, recognition and support scores significantly decreased by 0.09 Likert points (95% CI -0.14, -0.04; P=0.001). Age, work experience, gender, and perception of fairness of salary has no significant association with recognition and support.

Discussion

The extent of overall motivation in respondents across regions and cadres was generally high, but with some variation across regions and other characteristics. The motivation domains identified in a three-factor factor analysis were effort in personal and altruistic goals; pride and personal satisfaction; and recognition (financial and managerial) support.

Our finding of high overall motivation is consistent with several other studies. For example, a study conducted in West Amhara, Northwest Ethiopia showed that 59% of care providers working in hospitals were motivated (16). In West Shewa Zone of Oromia region, the overall mean motivation score of health professionals from three hospitals was 64% (3). In this study, motivation varied significantly from region to region, which previous smaller studies have not been able to explore. This variation is consistent with a study from Kenya that also found motivational factors varied from region to region (11). SNNPR had the lowest motivation, consistent with a study done among health professionals in SNNPR's Gedeo zone where only 17% of respondents were highly motivated. This could need further exploratory studies (9).

Formatted: Font: (Default) Calibri, Highlight

Formatted: Highlight

Formatted: Highlight

Personal and altruistic goals of health providers were significantly lower among participants from Oromia and SNNPR compared to those from the Amhara region. Average job satisfaction is strongly associated with high efforts in personal and altruistic goal.

Motivation related to pride and personal satisfaction was significantly high among participants older than 30 years of age as compared to those 24 years and younger. This study was consistent with studies from Central Ethiopia and South Ethiopia where age was a major predictor of motivation; as age increased, motivation to do their job increased (9,12). Similar studies in Burkina Faso, Ghana and Tanzania showed that age has a significant effect on motivation (26). Pride and personal satisfaction factor were significantly high among participants whose job satisfaction was high as of different studies in Ethiopia explains (27).

Recognition and support were significantly low among participants from SNNPR and Oromia region and was associated with low motivation. This could be due to instability within the system or the lack of capacity to supervise the health care workers located in a large geographical area. Among participants with increased average leave, recognition support was significantly low. This may explain why participants who were taking leave more were less motivated. Recognition and support were significantly higher among health care providers than among HEWs. Having more recognition (financial and managerial) support was a positive predictor for job satisfaction and motivation (8,28,29).

A strength of this study was that data was collected from four (Amhara, Oromia, SNNPR, Tigray) major regions of Ethiopia where most of the country's population live and the regional variation of motivation was studied at the same time, and the health care workers and health extension workers motivation were compared, in addition this study addressed computing multiple associated factors with the overall motivation and three motivation

Formatted: Highlight

Formatted: Highlight

Formatted: Highlight

Formatted: Highlight

Field Code Changed

Formatted: Highlight

Formatted: Highlight

factors identified during factor analysis. Nevertheless, limitations remain. Motivation was self-reported and may therefore be subject to acceptability biases in the face-to-face interviews conducted. Knowledge that interviews were conducted by interviewers from a public university and evaluating an IHI programme may have biased responses. The skilled care provider sample was too small to conduct a subgroup analysis among specific groups of interest such as doctors or midwives. Region-level analyses may have been underpowered to variation in motivation, particularly in the presence of heterogeneity in motivation within regions, for example by cadre, or other unobserved variables. The sampling strategy was not optimized to account for or detect potentially important heterogeneity between regions, urban and rural areas, or QI programme areas, which may further reduce power to detect across-region variation. Qualitative work could have been conducted alongside quantitative data collection to understand why variation exists in motivation between regions and personal characteristics. Finally, we were unable to assess the link between health worker motivation and the quality of care provided which remains under-researched.

Formatted: Highlight

Conclusion

Motivation was high among the respondents from the four regions. Key factors associated with motivation were region, age, cadre type, workload, job satisfaction, long leave days and work experience. Further studies are needed to explore the reason for variation in motivation across regions and cadre type.

Acknowledgements

We are grateful to all participants for their time in completing various study components. Excellent research assistance in data collection was provided by Teklit Grum, Tsegaab

Temesgen, Zelalem Tedla, Saba Shiferaw, Duresa Endale, Abeba Hailemelekot, Worknesh Daba, Hasna Musema, Selamawit Herpa, Tigist Abera, Samuel Ayele, and Aniley Dagife. The QI program is funded by the Bill and Melinda Gates Foundation and Margaret A. Cargill Philanthropies. We thank our colleagues from Addis Continental Institute of Public Health who provided insight and expertise that greatly assisted with the analysis and write-up of this research. We are especially indebted to Prof. Alemayehu Worku and Dr Walelign Worku for their tireless support in the analysis of the data. We are also grateful to Jane Roessner for her valuable comments on an earlier version of the manuscript and Naomi Fedna for proofreading the final version to fit journal requirement.

Funding

This work was supported by IDEAS—Informed Decisions for Actions to improve maternal and new-born health (http://ideas.lshtm.ac.uk), which is funded through a grant from the Bill & Melinda Gates Foundation to the London School of Hygiene & Tropical Medicine. (Gates Global Health Grant Number: OPP1149259). The funder of this study had no role in the study's design or conduct, data collection, analysis, or interpretation of results, writing of the paper, or decision to submit for publication.

Author's contributions

Conceptualization: Mehiret Abate, Mathew Quaife, Gareth Parry

Data curation: Mehiret Abate, Matthew Quaife

Formal analysis: Mehiret Abate, Matthew Quaife

Investigation: Dorka Woldesenbet, Abiy Seifu Estifanos, Abera Biadgo, Befikadu Bitewulign, Haregeweyni Alemu, Zewdie Mulissa, Mehiret Abate

Methodology: Mehiret Abate, Matthew Quaife, Gareth Parry

Project administration: Mehiret Abate, Hema Magge, Abiyou Kiflie, Abera Biadgo, Befikadu Bitewulign, Haregeweyni Alemu, Mehiret Abate, Zewdie Mulisa

Resources: Hema Magge, Abiyou Kiflie, Matthew Quaife

Supervision: Matthew Quaife, Mehiret Abate, Gareth Parry, Hema Magge,

Writing – original draft: Mehiret Abate.

Writing – review & editing: Mehiret Abate, Yakob Semagn, Abera Biadgo, Befikadu Bitewulign, Haregeweyni Alemu, Zewdie Mulisa, Dorka woldesenbet, Abiy Seifu Estifanos, Abiyou Kiflie, Hema Magge, Gareth Parry, Matthew Quaife

References

1. Franco LM, Bennett S, Kanfer R. Health sector reform and public sector health worker motivation: a conceptual framework. Social Science & Medicine. 2002 Apr;54(8):1255–66.

2. Mosadeghrad AM. Factors Influencing Healthcare Service Quality. International Journal of Health Policy and Management. 2014;3(2):77–89.

3. Dagne T, Beyene W, Berhanu N. Motivation and Factors Affecting It among Health Professionals in the Public Hospitals, Central Ethiopia. Ethiopian Journal of Health Sciences. 2015 Jul 27;25(3):231.

4. Toode K, Routasalo P, Suominen T. Work motivation of nurses: A literature review. International Journal of Nursing Studies. 2011 Feb 1;48(2):246–57.

5. Franco LM, Bennett S, Kanfer R, Stubblebine P. Determinants and consequences of health worker motivation in hospitals in Jordan and Georgia. Soc Sci Med. 2004 Jan;58(2):343–55.

6. Kumar P, Mehra A, Inder D, Sharma N. Organizational commitment and intrinsic motivation of regular and contractual primary health care providers. J Family Med Prim Care. 2016 Mar;5(1):94–100.

7. Mbilinyi, D., Daniel, M.L. & Lie, G.T. Health worker motivation in the context of HIV care and treatment challenges in Mbeya Region, Tanzania: A qualitative study. *BMC Health Serv Res* **11,** 266 (2011).https://doi.org/10.1186/1472-6963-11-266

8. Willis-Shattuck M, Bidwell P, Thomas S, Wyness L, Blaauw D, Ditlopo P. Motivation and retention of health workers in developing countries: a systematic review. BMC Health Serv Res. 2008 Dec 4;8:247.

9. Berhanu N, Ereso BM, Nebeb GT. Job Motivation and Associated Factors Among Health Care Professionals Working in Public Health Facilities of Gedeo Zone, Southern Ethiopia. Clinical Medicine Research. 2017 Feb 28;6(1):9.

10. Negussie N. Relationship Between Rewards and Nurses' Work Motivation in Addis Ababa Hospitals. Ethiop J Health Sci. 2012 Jul;22(2):107–12.

11. Ojakaa D, Olango S, Jarvis J. Factors affecting motivation and retention of primary health care workers in three disparate regions in Kenya. Human Resources for Health. 2014 Jun 6;12(1):33.

12. Beyene W. Work Motivation and Factors affecting it among Health Professionals in West Shoa Zone Public Hospitals, Oromia Region, Central Ethiopia. Ethiop J Health Sci. 2015 Jul 1;25:231–42.

13. Mbindyo, P., Gilson, L., Blaauw, D. *et al.* Contextual influences on health worker motivation in district hospitals in Kenya. *Implementation Sci* **4,** 43 (2009).https://doi.org/10.1186/1748-5908-4-43

14. Lunenburg FC. Self-Efficacy in the Workplace: Implications for Motivation and Performance. International journal of Management, Business and administration.2011;14(1):1-6.

15. Alhassan RK, Spieker N, van Ostenberg P, Ogink A, Nketiah-Amponsah E, de Wit TFR. Association between health worker motivation and healthcare quality efforts in Ghana. Human Resources for Health. 2013 Aug 14;11(1):37.

16. Weldegebriel Z, Ejigu Y, Weldegebreal F, Woldie M. Motivation of health workers and associated factors in public hospitals of West Amhara, Northwest Ethiopia. *Patient Prefer Adherence*. 2016;10:159-169. Published 2016 Feb 15. doi:10.2147/PPA.S90323

17. Selamu, M., Thornicroft, G., Fekadu, A. *et al.* Conceptualisation of job-related wellbeing, stress and burnout among healthcare workers in rural Ethiopia: a qualitative study. *BMC Health Serv Res* **17,** 412 (2017).https://doi.org/10.1186/s12913-017-2370-5

18. Biadgo A, Legesse A, Estifanos AS, Singh K, Mulissa Z, Kiflie A, et al. Quality of maternal and newborn health care in Ethiopia: a cross-sectional study. BMC Health Serv Res. 2021 Dec;21(1):679.

19. Strachan DL, Källander K, Nakirunda M, Ndima S, Muiambo A, Hill Z, et al. Using theory and formative research to design interventions to improve community health worker motivation, retention and performance in Mozambique and Uganda. Hum Resour Health. 2015 Apr 30;13:25.

20. Chandler CIR, Chonya S, Mtei F, Reyburn H, Whitty CJM. Motivation, money and respect: A mixed-method study of Tanzanian non-physician clinicians. Social Science & Medicine. 2009 Jun 1;68(11):2078–88.

21. Borghi J, Lohmann J, Dale E, Meheus F, Goudge J, Oboirien K, et al. How to do (or not to do)… Measuring health worker motivation in surveys in low- and middle-income countries. Health Policy Plan. 2018 Mar;33(2):192–203.

22. Ferguson, E. and T. Cox,. Exploratory factor analysis: A users' guide. International journal of selection and assessment. 1993;1(2):84–94.

23. Hilari, K. et. al. Stroke and aphasia quality of life scale-39 (SAQOL-39) evaluation of acceptability, reliability, and validity. Stroke, . 2003;34(8):1944–50.

24. Akinwande, M.O., Dikko, H.G. and Samson, A. (2015) Variance Inflation Factor: As a Condition for the Inclusion of Suppressor Variable(s) in Regression Analysis. Open Journal of Statistics, 5, 754-767.

26. Prytherch H, Leshabari MT, Wiskow C, Aninanya GA, Kakoko DCV, Kagoné M, et al. The challenges of developing an instrument to assess health provider motivation at primary

care level in rural Burkina Faso, Ghana and Tanzania. Global Health Action. 2012 Dec;5(1):19120.

27. Hotchkiss DR, Banteyerga H, Tharaney M. Job satisfaction and motivation among public sector health workers: evidence from Ethiopia. Hum Resour Health. 2015 Dec;13(1):83.

28. Daneshkohan A, Zarei E, Mansouri T, Maajani K, Ghasemi MS, Rezaeian M. Factors Affecting Job Motivation among Health Workers: A Study From Iran. Global Journal of Health Science. 2015;7(3):8.

29. Deschamps C, Rinfret N, Lagacé MC, Privé C. Transformational Leadership and Change: How Leaders Influence Their Followers' Motivation Through Organizational Justice. J Healthc Manag. 2016 Jun;61(3):194–213.

Supporting information

S1 Questionnaire. Questionnaire used for data collection in ODK file was prepared in both local (Amharic) and English language.

S2 Appendix 1. Table A1. Results from ordered logit model showing factors associated with overall motivation among participants form four regions April 15 – May 10, 2018

S3 Appendix 2. Table A2. Results from ordered logit model showing association between motivation factors and demographic and structural factors among participants from four regions April 15 – May 10,2018

S4 The datasets used and/or analysed during the current study DOI (https://doi.org/10.17037/DATA.00001565) will be available from London School of Hygiene and Tropical Medicine.

Feb 16, 2022

Manuscript PONE-D-21-09235

Response to reviewers

Dear Editor in Chief

Thank you so much for the opportunity you have given us to re-submit a revised manuscript entitled "Key factors influencing motivation among health extension workers and health care professionals in four regions of Ethiopia: a cross sectional study." for consideration for publication in PLOS ONE.

We appreciate the time and effort you and the reviewers dedicated to providing feedback to our manuscript and are grateful for the insightful comments on and valuable improvements made to our paper.

We have incorporated most of the suggestions made by the reviewer. Those changes are highlighted with yellow color within the revised manuscript with track changes. Please see below a blue colored point-by-point response to the reviewers' comments and concerns. Please note that all page numbers and line numbers refer to the revised manuscript.

Thank you so much once again!

Reviewer #1 no comment

Reviewer# 2: Key factors influencing motivation among health extension workers and health care professionals in four regions of Ethiopia: a cross-sectional study.

I am excited to get the opportunity to review the manuscript mentioned above. In general, the manuscript is a well-stated and essential area for the developing country. However, before accepting publication, the authors should consider the following issues.

Abstract:

Include the objective of the study in the abstract section. Make sure to use the same abstract in the system and main manuscript. The abstract in the main manuscript has no objectives, while the abstract in the system contains it.

Authors Response: thank you for pointing out this. We have included the objective on page 2 line 26-27 and it reads as *’Objective this study sought to determine the extent and variation of health professionals’ motivation alongside factors associated with motivation.’*

What does non-patient-facing health system staff mean? Who are they?

Authors response: thank you for asking, non-patient facing health system staffs are professionals working in different structures of a health system. It is stated on the abstract on page 2 line 32-33 as *’non-patient facing health system staff representing case team leaders, facility and district heads, directors, and officers (n=81).’*

Please give space between a statement and citation throughout the manuscript.

Authors response: Thank you for the comment, corrected accordingly as shown on line 50, 53, 55, 65, 72, 106, 109, 188, 322, 325, 332 and 343.

Line 65 -66

What was the finding of the study? Why did the author explain these studies?

Authors response: Thank you, the study assessed motivation and retention of health care workers and indicated importance of financial incentives on health workers motivation. In our study, there are different factors associated with motivation including financial incentives and revealed that financial and managerial support were significantly associated with health professionals job satisfaction, region, leave days and job title as specified on the result section on page 22 line 300 -310. These also further explained on the discussion section on page 24 on line 341 - 343 as *‘Recognition and support were significantly higher among health care providers*

than among HEWs. Having more recognition (financial and managerial) support was a positive predictor for job satisfaction and motivation.'

Even though the sample size is smaller, it includes four regions (Amhara, Oromo, SNNPR, and Tigray), which is good and improves the representativeness of the finding. But, what is your justification for including these? Why not Harari, Gambella.

Authors response: we believed that the four regions are home to more than 81% of the population in Ethiopia and accommodates most health facilities and health workers. These regions are highly diverse population and have different geographic characteristics improving representativeness as stated on page 6, line number 100 - 102.

Table 5:

Association between motivation factors and 271 demographic and structural factors Use consistent decimal numbers throughout the document. Example region: (-0.21, 0.006) 0.064

Authors response: Thank you for pointing this out, the decimal numbers corrected accordingly throughout the document. On page number 13 table 2 (region _Amhara %), on page number 17-line number 249, on page number 18 Table 4 perceived gross salary fair (p value), on page number 19 in line number 270, on page number 20-21 table 5 gender factor 2 (coef.), region factor 2 (coef.), factor 2 region (95%CI), age>30 factor1 (95%CI), work experience factor2 (coef.), factor3 job title (95%CI), on page number 22 in line number 299.

Discussion session

Page 22 and line 301-302: add the figure with confidence interval.

Authors response: thank you for pointing out, we have included the interval accordingly it reads as *'the mean score of recognition and support was 1.77 (95% CI 1.72,1.83; P=0.001)'* on page 22 line 299.

Page 22 and line 303: delete one "factor".

Authors response: thank you deleted accordingly.

Page 22 and lines 303 - 314: How do you think your finding is different from the previous one? Consider this comment for all results?

Authors response: line number 303-314 our study finding "*motivation varied from region to region''* is different in its scope that the study was conducted in four big regions and regional variation of motivation was studied at the same time. In addition, the health care workers motivation and health extension workers motivation were compared, and this study is different in computing multiple characteristics with the overall motivation and three motivation factors.

The three factors also indicated variation with associated factors (region, age, leave days, job satisfaction, perceived gross salary, workload…) as on page number 17-line number 249-257, on page number 19-line number 271-280, and on page number 22-line number 287 – 295.

This has been addressed in the manuscript discussion section on page 24 and line number 345-349 stated as *'A strength of this study was that data was collected from four (Amhara, Oromia, SNNPR, Tigray) major regions of Ethiopia where most of the country's population live and the regional variation of motivation was studied at the same time, and the health care workers and health extension workers motivation were compared, in addition this study addressed computing multiple associated factors with the overall motivation and three motivation factors identified during factor analysis.'*

What is the limitation of your study?

Authors response: thank you for this question, we have already stated a number of limitations in the manuscript on page24 & 25, line number 348-361:

'Motivation was self-reported and may therefore be subject to acceptability biases in the face-to-face interviews conducted. Knowledge that interviews were conducted by interviewers from a public university and evaluating an IHI programme may have biased responses. The skilled care provider sample was too small to conduct a subgroup analysis among specific groups of interest such as doctors or midwives. Region-level analyses may have been underpowered to variation in motivation, particularly in the presence of heterogeneity in motivation within regions, for example by cadre, or other unobserved variables. The sampling strategy was not optimized to

account for or detect potentially important heterogeneity between regions, urban and rural areas, or QI programme areas, which may further reduce power to detect across-region variation. Qualitative work could have been conducted alongside quantitative data collection to understand why variation exists in motivation between regions and personal characteristics. Finally, we were unable to assess the link between health worker motivation and the quality of care provided which remains under-researched.'
