# Supplementary material for: Key factors influencing motivation among health extension workers and health care professionals in four regions of Ethiopia: A cross-sectional study
Source: PLoS One. 2022 Sep 30;17(9):e0272551. doi: 10.1371/journal.pone.0272551 (PMC9524639; doi:10.1371/journal.pone.0272551)
Supplement: Attachment — Submitted filename: Concern.docx [file pone.0272551.s004.docx]

This is the manuscript titled "Key factors influencing motivation among health extension workers and health care professionals in four regions of Ethiopia: a cross-sectional study." by Mehiret A. et al.

Firstly, I would like to thank the authors for this work. This is an important study that attempts to address Key factors influencing motivation among health extension workers and health care professionals in four regions of Ethiopia. The study sheds light on the challenges of ensuring quality care through motivated health extension workers and health care professionals. All countries worldwide have signed up to the United Nations Sustainable Development Goals (UNSDGs) and have committed to achieving universal health coverage, including financial risk protection, access to quality essential healthcare services, and access to safe, adequate, quality, affordable essential medicines and vaccines for all. However, health care coverage in Ethiopia is still a big concern, which requires addressing all components. Therefore, I am glad to be a reviewer of this work.

As a general comment, the authors should improve the language, introduction, and discussion section.

Specific comments

**Abstract**

- Background: The authors need to justify more the reason that initiates them to study this topic. Make sure to show the gap clearly, and include the objective also.
- Method: the authors should explain the critical components of an abstract such as study period, study setting,

**Introduction:**

- The introduction was written pretty well but still needs to show the consequences of inadequate health professionals and health extension worker motivation on the population health in general, such as death, a dropout from service, prolonged hospitalization, poor attitude to health facilities visit and soon.

**Methods**

- Line page 10: check for typo-error such as … established through a “Scree” test and multiple…
- Page 10, on lines 179 to 181, the authors mentioned that “the average job satisfaction was assessed by re-coding the 5-point Likert scale ranging from most satisfied with their job (1) to least satisfied (5) as a continuous variable.” Why did they need to consider scale one as most satisfied and scale five least? The revers will be more logical. Can you please explain this? Most studies and scholars use 5 as the most satisfied, while 1 is the least satisfied.

**Result sections**

1. Table titles are not self-explanatory. Table/figure titles must be self-explanatory, which means every audience should be understood what the figure/table contains.
2. The number of the total participants is not consistently explained in the document. For example, the authors told us that only 397 participants were provided their responses. However, they explained 401 respondents in the table. Similarly, the authors should re-check that the sum of all participants is similar to the total respondents. E.g., in Table 2: region, the sum of 107, 106, 137, 50 is 400, which is not equal to the total participants explained by the title. Same table, data for age: the sum of 90, 232, 75 is …. Check other also.
3. Page 16, line 234/5… most demotivating factor mentioned by 29% … add the confidence interval.
4. What are your criteria for selecting a reference group in the regression analysis?

**Discussion section**

1. What is the reason or reference to say motivation among HEW is high?
2. What is the possible reason health extension workers get motivated in the Amhara region compared to the SNNP? This might be good to scale up to other regions.

Recommendation

- Based on your finding, what do you suggest/recommend for the concerned body?
