# Supplementary material for: Key factors influencing motivation among health extension workers and health care professionals in four regions of Ethiopia: A cross-sectional study
Source: PLoS One. 2022 Sep 30;17(9):e0272551. doi: 10.1371/journal.pone.0272551 (PMC9524639; doi:10.1371/journal.pone.0272551)
Supplement: Attachment — Submitted filename: Response to reviewers.docx [file pone.0272551.s005.docx]

September 26, 2021

**Manuscript PONE-D-21-09235**

**Response to reviewers**

Dear Editor in Chief

# Thank you so much for the opportunity you have given us to re-submit a revised manuscript entitled “Key factors influencing motivation among health extension workers and health care professionals in four regions of Ethiopia: a cross sectional study.” for consideration for publication in PLOS ONE.

We appreciate the time and effort you and the reviewers dedicated to providing feedback to our manuscript and are grateful for the insightful comments on and valuable improvements made to our paper.

We have incorporated most of the suggestions made by the reviewers. Those changes are highlighted with yellow color within the revised manuscript with track changes. Please see below a blue colored point-by-point response to the reviewers’ comments and concerns. Please note that all page numbers and line numbers refer to the revised manuscript.

Thank you so much once again!

**Reviewers’ comments to the Authors**

**Comment from academic editor**

1. Row 127: you mention the "woredas", I am suspecting it is something like regions? Please either give the interpretation for international readers that are not familiar with local terms or substitute with the international term.

**Authors response:** thank you. The author substituted “Woreda” with known equivalent term “District” as indicated in line 128,129,131,133,140, 144, 145,147 and 149.

**Comment from academic editor**

2. Row 175-176: you mention that you utilise regression models. Please elaborate more. Do you estimate OLS models (I am suspecting yes, based on my comment below) and why you choose the OLS regression since your dependent is qualitative?

**Authors response:** thank you. Yes, the Authors used OLS models to explore determinants of motivation level. We have now changed methods text to read (additions in bold): “We explored the association of overall motivation and with the motivation factors identified with overall job satisfaction and several demographic and structural factors including gender, location, cadre, age, perceived gross salary, work experience, using univariate and **multivariate ordinary least squares regression models, and show ordered logistic regression model results in the appendix** 1&2 mentioned in line 184 and supporting information S2 & 3 as in line 507 & 509.

Ordinary least squares models are able to give unbiased coefficients for ordered categorical outcomes, though we accept the point that interpretation is difficult, and this is non-standard in some fields. As above, therefore, we have fitted ordered logistic regression models to the data and present results in the appendix. The results of these models are consistent with OLS results with respect to sign, significance, and magnitude of independent variable coefficients.

**Comment academic editor**

3. Rows 179-181: you state that the job satisfaction scores are recoded to transform the variable to a continuous one (if I understand correctly). How do you do that and what is the rationale to justify this transformation?

**Author response:** We did not transform the variables; we simply took categorical Likert Scale responses ranging from most satisfied in their job (5) to least satisfied (1) and used this as dependent variable (as described in line 189 - 190).

**Comment from academic editor**

4. Rows 240-242: you state that "... where an increase of 1 in job 241 satisfaction was associated with an increase in motivation of 0.23 points". I would discourage you from using such strong assumptions. For one, the models estimated do not necessarily imply causation and secondly, both variables are qualitative, so such an interpretation has no logical meaning. I would advise estimating logit/ordered logit models and assess the probability to respond a higher/lower score base on certain characteristics of respondents, since it would make more sense to do that. Please remove such quantitative interpretations from the results and the discussion.

**Author response:** Thank you for pointing this out, and we agree interpretation is difficult here. We have changed these results to describe sign and significance rather than magnitude as suggested on the following lines:

line 250-252 reads as “Average job satisfaction was strongly associated with higher motivation (P=0.001).”

line 272-274 reads as “From the results of the regression analysis summarized in Table 5, average job satisfaction was strongly associated with higher personal and altruistic goals score (P=0.001).”

line 293-295 reads as “Average job satisfaction is strongly associated with high pride and personal satisfaction (P=0.001)”

line 308-310 reads as “Average job satisfaction is strongly associated with higher recognition and support score (P=0.001).”

**Comment from academic editor**

5. Please interpret your findings with caution: state the problems of establishing causality and remove statements that suggest otherwise (for example rows 316-317).

**Author response:** Thank you for pointing this out, and as above we have changed this language throughout. Sentences corrected based on the reviewer comments as in line 330-332: reads as “Average job satisfaction is strongly associated with high efforts in personal and altruistic goal.”

**Comment from reviewer #1**

It is a commendable study to have collected data from four different regions of Ethiopia and different cadres of health workers, including HEWs. The addition of the following information would improve the quality of this manuscript.

**Author response:** Thank you for reviewing the manuscript, and for your comments which have improved the paper.

**Comment from Reviewer #1**

1. References
   In the Introduction/Methods sections, it is better to cite the reference (e.g. line 60 of page 3, line 66 of page 4 [to add additional reference as you state "three studies" in the beginning], line 100 of page 5, and Source column of Table 1 of page 6 [rather than describing which study, cite the reference number])

**Authors response:** thank you for pointing this out. Reference included in the introduction and Method section, and on table 1 source column as on line 66, 75,108, 114&15 table 1 of page 7 line.

**Comment from Reviewer #1**

2. Name of the location: "Degua Temben" line 129 on page 8, might be Degua "Tembien"

**Author response:** thank you it is a typing error corrected as Degua “Tembien” as on line 130

**Comment from Reviewer #1**

3. Sampling One of the biggest concerns of this manuscript was the sampling. Please kindly respond to the following points:
   a. Does the sample size for this study have enough statistical power to discern the regional difference of motivation among four regions, especially having Tigray only 50 participants?

**Author response:** Thank you for pointing this out, this is a good point. We found this as a typing error where the participants from Tigray were74 as shown in page 13 of table 2, in addition we have added the following text in the limitations section of the discussion in line 355-358:

“Region-level analyses may have been underpowered to variation in motivation, particularly in the presence of heterogeneity in motivation within regions, for example by cadre, or other unobserved variables.”

b. Please state how the target of 50 respondents was set.

**Author response:** Thank you for allowing us to clarify this. We have added the following to the methods section as shown in line152-155:

“A target sample size of 50 respondents per region was chosen, based on the primary research question of assessing changes in motivation as measured by Likert scale questions, in line with a rule of thumb in exploratory factor analysis that 50 participants per cluster is a reasonable sample size to detect differences across clusters (20).”

c. Did the authors consider cluster random sampling taking population size, rural/urban differences, intervention/non-intervention areas of IHI into consideration? If not, why not?

**Author response:** Thank you for pointing this out. Cluster random sampling was not possible due to operational constraints, but the reviewer is correct to suggest that this would have allowed us to consider variation by potentially important observable characteristics in the sampling design. We have added the following to the limitations section to acknowledge this as shown in line 358-360:

“The sampling strategy was not optimized to account for or detect potentially important heterogeneity between regions, urban and rural areas, or QI program areas, which may further reduce power to detect across-region variation.”

**Comment from reviewer#2**

This is the manuscript titled "Key factors influencing motivation among health extension workers and health care professionals in four regions of Ethiopia: a cross-sectional study." by Mehiret A. et al.
Firstly, I would like to thank the authors for this work. This is an important study that attempts to address Key factors influencing motivation among health extension workers and health care professionals in four regions of Ethiopia. The study sheds light on the challenges of ensuring quality care through motivated health extension workers and health care professionals. All countries worldwide have signed up to the United Nations Sustainable Development Goals (UNSDGs) and have committed to achieving universal health coverage, including financial risk protection, access to quality essential healthcare services, and access to safe, adequate, quality, affordable essential medicines and vaccines for all. However, health care coverage in Ethiopia is still a big concern, which requires addressing all components. Therefore, I am glad to be a reviewer of this work.

As a general comment, the authors should improve the language, introduction, and discussion section.

**Author response:** Thank you. we are very much grateful to the reviewers for their close reading of the paper and constructive comments which we believe to improve our paper.

**Comment from reviewer #2**

1. Background: The authors need to justify more the reason that initiates them to study this topic. Make sure to show the gap clearly and include the objective also.

**Author Response:** thank you for pointing this out. The reviewer is correct, and we have included the gap and objective.

The revised text on background section of Abstract on line 24-27 of Page2 reads as “Low health care workers motivation can be associated with poor health care quality and client experience, long patient waiting time, non-attendance, and poor clinical outcome. Thus, this study sought to determine the extent and variation of health professionals’ motivation alongside factors associated with motivation.”

**Comment from reviewer #2**

2. Method: the authors should explain the critical components of an abstract such as study period, study setting,

**Author response:** Thank you for pointing this out. Study time and setting incorporated on the method section of the Abstract line 27-30 of page2

“We conducted a facility based cross-sectional study among health extension workers (HEWs) and health care professionals in four regions: Amhara, Oromia, South nations, and nationalities people’s region (SNNPR) and Tigray from April 15 to May 10, 2018.”

**Comment from reviewer #2**

The introduction was written pretty well but still needs to show the consequences of inadequate health professionals and health extension worker motivation on the population health in general, such as death, a dropout from service, prolonged hospitalization, poor attitude to health facilities visits and soon.

**Authors response.** Thank you for the insight included the consequences of low motivation to service on page4/5 line 70-73 reads as “Low health care workers motivation is a major contributor to the poor health service quality and client experience, long waiting time, nonattendance, and unofficial fee charges and poor clinical outcome.”

**Comment from reviewer #2**

**Method section**

1. Line page 10: check for typo-error such as … established through a “Scree” test and multiple…

**Authors response:** Thank you for pointing this out. Word corrected as “scree test” on line 177 of page 11

2. Page 10, on lines 179 to 181, the authors mentioned that “the average job satisfaction was assessed by re-coding the 5-point Likert scale ranging from most satisfied with their job (1) to least satisfied (5) as a continuous variable.” Why did they need to consider scale one as most satisfied and scale five least? The revers will be more logical. Can you please explain this? Most studies and scholars use 5 as the most satisfied, while 1 is the least satisfied.

**Author Response:** Thank you for pointing this out. The Author recognized as an error and corrected on page 12 of line 188-190 Reads as “Average job satisfaction was assessed by re-coding the 5-point Likert scale ranging from least satisfied with their job (1) to most satisfied (5) as a continuous variable.”

**Comment from reviewer #2**

**Result section**

1. Table titles are not self-explanatory. Table/figure titles must be self-explanatory, which means every audience should be understood what the figure/table contains.

**Author response:** thank you for pointing this out. Table & figure titles Corrected.

The corrected table and titles read as follows:

**Table 1. Motivation questions included in survey, source, and domain from April 15-May 10, 2018** (Line 114 on page7)

**Table 2. Background characteristics of participants from four regions, (n=397), April 15-May 10, 2018** (Page13, line203)

**Figure 1. Plot of responses to motivational survey items among participants from four regions of Ethiopia, April 15-May 10, 2018** (Page 14 of line211)

**Table 3. Exploratory factor analysis results showing the factor loadings by individual items April 15- May 10, 2018** (line235, page16)

**Table 4. Factors associated with overall motivation among participants form four regions April 15- May 10, 2018** (line263 of page 18)

**Table 5. Association between motivation factors and demographic and structural factors among participants from four regions April 15- May 10, 2018.** (Line 282 of page 20)

2. The number of the total participants is not consistently explained in the document. For example, the authors told us that only 397 participants were provided their responses. However, they explained 401 respondents in the table. Similarly, the authors should re-check that the sum of all participants is similar to the total respondents. E.g., in Table 2: region, the sum of 107, 106, 137, 50 is 400, which is not equal to the total participants explained by the title. Same table, data for age: the sum of 90, 232, 75 is …. Check other also.

**Author response:** The authors corrected that the total respondents completed the survey n= 397 as shown in the manuscript result section and table 2 of page 13 on line 203.

3. Page 16, line 234/5… most demotivating factor mentioned by 29% … add the confidence interval.

**Authors response:** Confidence interval included on page 17 line 245, read as “The most demotivating factor mentioned by 29% participants was workload (95% CI 34% - 39%).”

4. What are your criteria for selecting a reference group in the regression analysis?

**Author’s response:**

The criteria for selecting the reference group were the random Alphabet order (Amhara).

**Comment from reviewer #2**

**Discussion**

1. What is the reason or reference to say motivation among HEW is high?

**Author response:** we explain that of the respondents completed the study, 61% of them were motivated to do their job. We did not mention specific cadre type (HEW).

2. What is the possible reason health extension workers get motivated in the Amhara region compared to the SNNP? This might be good to scale up to other regions.

**Author response:** From regression analysis the recognition and support motive among respondent were significantly lower in SNNPR & OROMIA as compared to Amhara region. This may explain that recognition and support in this region high. As studies show that recognition is a major predictor for motivation.

3. Based on your finding, what do you suggest/recommend for the concerned body?

**Authors response:** We have edited the manuscript accordingly on page 25 in line 368-369

“Further studies are needed to explore the reason for variation in motivation across regions and cadre type.”

**Response to Supporting information:**

#1Questionnnaire: All variables included in the analysis are explained fully in the text and figures, and we have sought to ensure that replication would be possible from these. Questionnaire used for data collection was done with ODK as in line 164-165. We will provide the ODK file which was prepared both in local language (Amharic) and English language up on acceptance and reasonable request.

#2 Data: The datasets used and/or analyzed during the current study DOI ( [https://doi.org/10.17037/DATA.00001565](https://nam12.safelinks.protection.outlook.com/?url=https%3A%2F%2Fdoi.org%2F10.17037%2FDATA.00001565&data=04%7C01%7C%7C19600ed3e41b4796a5a808d984faa229%7Cae635716f1924ebca7c071136d785df2%7C0%7C0%7C637687034278790740%7CUnknown%7CTWFpbGZsb3d8eyJWIjoiMC4wLjAwMDAiLCJQIjoiV2luMzIiLCJBTiI6Ik1haWwiLCJXVCI6Mn0%3D%7C1000&sdata=a6RKR5d0Q830hjdOtlR7XOTz%2BY%2FTn4dVAUKkUk%2FFwqE%3D&reserved=0)) will be available from London School of Hygiene and Tropical Medicine.

#3 Pretest: The survey was piloted out of 19 district health office staff in December 2017. No changes were made to the survey between piloting and the final survey as it was understood well by participants, assessed through debriefing interviews after survey completion as shown in line141-144.
