# Supplementary material for: Key factors influencing motivation among health extension workers and health care professionals in four regions of Ethiopia: A cross-sectional study
Source: PLoS One. 2022 Sep 30;17(9):e0272551. doi: 10.1371/journal.pone.0272551 (PMC9524639; doi:10.1371/journal.pone.0272551)
Supplement: Attachment — Submitted filename: Response to reviewers.docx [file pone.0272551.s006.docx]

Feb 16, 2022

**Manuscript PONE-D-21-09235**

**Response to reviewers**

Dear Editor in Chief

Thank you so much for the opportunity you have given us to re-submit a revised manuscript entitled “Key factors influencing motivation among health extension workers and health care professionals in four regions of Ethiopia: a cross sectional study.” for consideration for publication in PLOS ONE.

We appreciate the time and effort you and the reviewers dedicated to providing feedback to our manuscript and are grateful for the insightful comments on and valuable improvements made to our paper.

We have incorporated most of the suggestions made by the reviewer. Those changes are highlighted with yellow color within the revised manuscript with track changes. Please see below a blue colored point-by-point response to the reviewers’ comments and concerns. Please note that all page numbers and line numbers refer to the revised manuscript.

Thank you so much once again!

**Reviewer #1** no comment

**Reviewer# 2:** Key factors influencing motivation among health extension workers and health care professionals in four regions of Ethiopia: a cross-sectional study.

I am excited to get the opportunity to review the manuscript mentioned above. In general, the manuscript is a well-stated and essential area for the developing country. However, before accepting publication, the authors should consider the following issues.

**Abstract:**

Include the objective of the study in the abstract section. Make sure to use the same abstract in the system and main manuscript. The abstract in the main manuscript has no objectives, while the abstract in the system contains it.

**Authors Response:** thank you for pointing out this. We have included the objective on page 2 line 26-27 and it reads as *’Objective this study sought to determine the extent and variation of health professionals’ motivation alongside factors associated with motivation.’*

What does non-patient-facing health system staff mean? Who are they?

**Authors response:** thank you for asking, non-patient facing health system staffs are professionals working in different structures of a health system. It is stated on the abstract on page 2 line 32-33 as *’non-patient facing health system staff representing case team leaders, facility and district heads, directors, and officers (n=81).’*

Please give space between a statement and citation throughout the manuscript.

**Authors response:** Thank you for the comment, corrected accordingly as shown on line 50, 53, 55, 65, 72, 106, 109, 188, 322, 325, 332 and 343.

**Line 65 -66**

What was the finding of the study? Why did the author explain these studies?

**Authors response:** Thank you, the study assessed motivation and retention of health care workers and indicated importance of financial incentives on health workers motivation. In our study, there are different factors associated with motivation including financial incentives and revealed that financial and managerial support were significantly associated with health professionals job satisfaction, region, leave days and job title as specified on the result section on page 22 line 300 -310. These also further explained on the discussion section on page 24 on line 341 - 343 as *‘**Recognition and support were significantly higher among health care providers than among HEWs. Having more recognition (financial and managerial) support was a positive predictor for job satisfaction and motivation.’*

Even though the sample size is smaller, it includes four regions (Amhara, Oromo, SNNPR, and Tigray), which is good and improves the representativeness of the finding. But, what is your justification for including these? Why not Harari, Gambella.

**Authors response:** we believed that the four regions are home to more than 81% of the population in Ethiopia and accommodates most health facilities and health workers. These regions are highly diverse population and have different geographic characteristics improving representativeness as stated on page 6, line number 100 - 102.

**Table 5:**

Association between motivation factors and 271 demographic and structural factors
Use consistent decimal numbers throughout the document. Example region: (-0.21, 0.006) 0.064

**Authors response:** Thank you for pointing this out, the decimal numbers corrected accordingly throughout the document. On page number 13 table 2 (region _Amhara %), on page number 17-line number 249, on page number 18 Table 4 perceived gross salary fair (p value), on page number 19 in line number 270, on page number 20-21 table 5 gender factor 2 (coef.), region factor 2 (coef.), factor 2 region (95%CI), age>30 factor1 (95%CI), work experience factor2 (coef.), factor3 job title (95%CI), on page number 22 in line number 299.

**Discussion session**

Page 22 and line 301-302: add the figure with confidence interval.

**Authors response:** thank you for pointing out, we have included the interval accordingly it reads as *‘the mean score of recognition and support was 1.77 (95% CI 1.72,1.83; P=0.001)*’on page 22 line 299.

Page 22 and line 303: delete one “factor”.

**Authors response:** thank you deleted accordingly.

Page 22 and lines 303 - 314: How do you think your finding is different from the previous one? Consider this comment for all results?

**Authors response:** line number 303-314 our study finding “*motivation varied from region to region’’* is different in its scope that the study was conducted in four big regions and regional variation of motivation was studied at the same time. In addition, the health care workers motivation and health extension workers motivation were compared, and this study is different in computing multiple characteristics with the overall motivation and three motivation factors.

The three factors also indicated variation with associated factors (region, age, leave days, job satisfaction, perceived gross salary, workload…) as on page number 17-line number 249-257, on page number 19-line number 271-280, and on page number 22-line number 287 – 295.

This has been addressed in the manuscript discussion section on page 24 and line number 345-349 stated as *‘**A strength of this study was that data was collected from four (Amhara, Oromia, SNNPR, Tigray) major regions of Ethiopia where most of the country’s population live and the regional variation of motivation was studied at the same time, and the health care workers and health extension workers motivation were compared, in addition this study addressed computing multiple associated factors with the overall motivation and three motivation factors identified during factor analysis.’*

What is the limitation of your study?

**Authors response:** thank you for this question, we have already stated a number of limitations in the manuscript on page24 & 25, line number 348-361:

‘*Motivation was self-reported and may therefore be subject to acceptability biases in the face-to-face interviews conducted. Knowledge that interviews were conducted by interviewers from a public university and evaluating an IHI programme may have biased responses. The skilled care provider sample was too small to conduct a subgroup analysis among specific groups of interest such as doctors or midwives. Region-level analyses may have been underpowered to variation in motivation, particularly in the presence of heterogeneity in motivation within regions, for example by cadre, or other unobserved variables. The sampling strategy was not optimized to account for or detect potentially important heterogeneity between regions, urban and rural areas, or QI programme areas, which may further reduce power to detect across-region variation. Qualitative work could have been conducted alongside quantitative data collection to understand why variation exists in motivation between regions and personal characteristics. Finally, we were unable to assess the link between health worker motivation and the quality of care provided which remains under-researched*.’
