# Supplementary material for: Key factors influencing motivation among health extension workers and health care professionals in four regions of Ethiopia: A cross-sectional study
Source: PLoS One. 2022 Sep 30;17(9):e0272551. doi: 10.1371/journal.pone.0272551 (PMC9524639; doi:10.1371/journal.pone.0272551)
Supplement: Attachment — Submitted filename: Response to reviewers.docx [file pone.0272551.s008.docx]

May 28, 2022

**Manuscript PONE-D-21-09235**

**Response to reviewers**

Dear Editor in Chief

Thank you so much for the opportunity you have given us to re-submit a revised manuscript entitled “Key factors influencing motivation among health extension workers and health care professionals in four regions of Ethiopia: a cross sectional study.” for consideration for publication in PLOS ONE.

We appreciate the time and effort you and the reviewers dedicated to providing feedback to our manuscript and are grateful for the insightful comments and valuable improvements made to our paper.

We have incorporated most of the suggestions made by the reviewer. Those changes are highlighted with yellow color within the revised manuscript with track changes. Please see below a blue colored point-by-point response to the reviewers’ comments and concerns. Please note that all page numbers and line numbers refer to the revised manuscript.

Thank you so much once again!

**Reviewer#1: No comment**

**Reviewer# 2:**

The abstract is not structured well. the authors need to restructure it clearly separating the important sections.

**Authors response:** Thank you. Yes, we separated all the important components of the abstract Background, Objectives, Methods, Results, and conclusion section without violating the PLOS ONE manuscript body submission guideline. The statement explained as

**“****Background:** Although Ethiopia has improved access to health care in recent years, quality of care remains low. Health worker motivation is an important determinant of performance and affects quality of care. Low health care workers motivation can be associated with poor health care quality and client experience, non-attendance, and poor clinical outcome. Objective his study sought to determine the extent and variation of health professionals’ motivation alongside factors associated with motivation.

**Methods:** We conducted a facility based cross-sectional study among health extension workers (HEWs) and health care professionals in four regions: Amhara, Oromia, South nations, and nationalities people’s region (SNNPR) and Tigray from April 15 to May 10, 2018. We sampled 401 health system workers: skilled providers including nurses and midwives (n=110), HEWs (n=210); and non-patient facing health system staff representing case team leaders, facility and district heads, directors, and officers (n=81). Participants completed a 30-item Likert scale ranking tool which asked questions across 17 domains. We used exploratory factor analysis to explore latent motivation constructs.

**Results:** Of the 397 responses with complete data, 61% (95% CI 56%-66%) self-reported motivation as “very good” or “excellent”. Significant variation in motivation was seen across regions with SNNPR scoring significantly lower on a five-point Likert scale by 0.35 points (P=0.003). The exploratory factor analysis identified a three-factors: personal and altruistic goals; pride and personal satisfaction; and recognition and support. The personal and altruistic goals factor varied across regions with Oromia and SNNPR being significantly lower by 0.13 (P=0.018) and 0.12 (P=0.039) Likert points respectively. The pride and personal satisfaction factor were higher among those aged >=30 years by 0.14 Likert scale points (P=0.045) relative to those aged between 19-24years.

**Conclusions:** Overall, motivation was high among participants but varied across region, cadre, and age. Workload, leave, and job satisfaction were associated with motivation.”

**Reviewer#2**

The method section needs to be restructured. Some important sections were still not included. Try to consider all necessary components.

**Authors response:** thank you, we included the necessary components “Study design” and moved the sentence under Materials and Methods sub section (study design) as stated in line 104-107 as “We conducted a facility based cross-sectional study among health extension workers (HEWs) and health care professionals in four regions: Amhara, Oromia, South nations, and nationalities people’s region (SNNPR) and Tigray from April 15 to May 10, 2018. “

In addition, on sub section (sampling and data collection) in line 131-133 reads as “We sampled 401 health system workers: skilled providers including nurses and midwives (n=110), HEWs (n=210); and non-patient facing health system staff representing case team leaders, facility and district heads, directors, and officers (n=81).”

**Reviewer#2**

Line136: Why purposive? How do you analyze data obtained from the two randomly and nonrandom?

**Authors response:** thank you. Purposive sampling was done for two Woreda which had simultaneous qualitative work ongoing in the broader QI evaluation (Page 144).

There was no evidence (before or after) analysis that key dimensions or results differed between randomly and non-randomly chosen woreda, so these data are pooled together. This analysis strategy was also taken in two previously published papers using this dataset:

Quaife, M., Estafinos, A. S., Keraga, D. W., Lohmann, J., Hill, Z., Kiflie, A., ... & Schellenberg, J. (2021). Changes in health worker knowledge and motivation in the context of a quality improvement programme in Ethiopia. *Health policy and planning*, *36*(10), 1508-1520.

Lamba, S., Arora, N., Keraga, D. W., Kiflie, A., Jembere, B. M., Berhanu, D., ... & Quaife, M. (2021). Stated job preferences of three health worker cadres in Ethiopia: a discrete choice experiment. *Health Policy and Planning*, *36*(9), 1418-1427.

**Reviewer#2**

Line 155, Why 50? Some regions contain larger number of the population Line 162

**Authors response:**

As stated on in line 163-166 “A target sample size of 50 respondents per region was chosen, based on the primary research question of assessing changes in motivation as measured by Likert scale questions, in line with a rule of thumb in exploratory factor analysis that 50 participants per cluster is a reasonable sample size to detect differences across clusters (21)”. Weighting the number of respondents by region size would not have led to more generalizable inference.

**Reviewer #2** Result section line 202, 203 Add frequency

**Authors response:**

Thank you included the frequency as on line 210-211. Reads as “Two hundred five (51%) of respondents had greater than 4 years of work experience, and (208, 52.39%) of the respondents were HEWs shown in Table 2.”

**Reviewer #2**

Page13, 18 and 21 in Table 2,4 & 5: (job title Other) who are this try to explain or mention all

**Authors response:** Thank you. We included all as Other (facility and district head, directors, and officers), and Leaders as case team leader, on page 13, 18, 21 in table 2, 4 and 5.

**Reviewer #2**

Discussion: How high is it? Where is the figure? What was your reference to conclude finding is high? How much is the variation among region and cadre?

**Authors response:**

Thank you for pointing out this. We corrected the wording as per the comment.

Our study has revealed the extent of motivation indicating 61% (majority of the participants were motivated to do their job). We stated as “Our study results indicated that more than 60% of HEW and health workers were motivated to do their job. Key factors identified to influence motivation were region, age, job title, work experience, job satisfaction, and leave days.”

We also already cited references from previous studies to compare with our study. Though, the studies were conducted among health professionals working in hospitals.

The study has also indicated the regional variation in general was underpowered as already mentioned as a limitation in line 367-373.
